# The H3K27me3 demethylase REF6 promotes leaf senescence through directly activating major senescence regulatory and functional genes in *Arabidopsis*

**DOI:** 10.1371/journal.pgen.1008068

**Published:** 2019-04-10

**Authors:** Xiaolei Wang, Jiong Gao, Shan Gao, Yi Song, Zhen Yang, Benke Kuai

**Affiliations:** 1 State Key Laboratory of Genetic Engineering, School of Life Sciences, Fudan University, Shanghai, China; 2 School of Life Sciences, Qilu Normal University, Jinan, China; 3 Ministry of Education Key Laboratory for Biodiversity Science and Ecological Engineering, Institute of Biodiversity Science, Fudan University, Shanghai, China; UT Austin, UNITED STATES

## Abstract

The roles of histone demethylation in the regulation of plant flowering, disease resistance, rhythmical response, and seed germination have been elucidated recently; however, how histone demethylation affects leaf senescence remains largely unclear. In this study, we exploited yeast one-hybrid (Y1H) to screen for the upstream regulators of *NONYELLOWING1* (*NYE1*), and identified RELATIVE OF EARLY FLOWERING6 (REF6), a histone H3 lysine 27 tri-methylation (H3K27me3) demethylase, as a putative binding protein of *NYE1* promoter. By *in vivo* and *in vitro* analyses, we demonstrated that REF6 directly binds to the motif CTCGYTY in *NYE1*/*2* promoters through its zinc finger domain and positively regulates their expression. Loss-of-function of REF6 delayed chlorophyll (Chl) degradation, whereas overexpression of *REF6* accelerated Chl degradation. Subsequently, we revealed that REF6 positively regulates the general senescence process by directly up-regulating *ETHYLENE INSENSITIVE 2* (*EIN2*), *ORESARA1* (*ORE1*), *NAC-LIKE*, *ACTIVATED BY AP3*/*PI* (*NAP*), *PYRUVATE ORTHOPHOSPHATE DIKINASE* (*PPDK*), *PHYTOALEXIN DEFICIENT 4* (*PAD4*), *LIPOXYGENASE 1* (*LOX1)*, *NAC DOMAIN CONTAINING PROTEIN 3* (*AtNAC3*), and *NAC TRANSCRIPTION FACTOR-LIKE 9* (*NTL9*), the key regulatory and functional genes predominantly involved in the regulation of developmental leaf senescence. Importantly, loss-of-function of REF6 increased H3K27me3 levels at all the target *Senescence associated genes* (*SAGs*). We therefore conclusively demonstrate that H3K27me3 methylation represents an epigenetic mechanism prohibiting the premature transcriptional activation of key developmentally up-regulated senescence regulatory as well as functional genes in *Arabidopsis*.

## Author summary

Leaves of higher plants start yellowing and subsequently die (senescence) at particular developmental stages as a result of both internal and external regulations. Leaf senescence is evolved to facilitate nutrient remobilization to young/important organs to meet their rapid development, and a large number of genes (*Senescence associated genes*, *SAGs*) are activated to regulate/facilitate the process. It has been intriguing how these genes are kept transcriptionally inactive to ensure an effective photosynthesis before the initiation of leaf senescence. Here, we reveal an epigenetic mechanism responsible for the prohibition of their premature transcription. We found that an H3K27me3 demethylase, RELATIVE OF EARLY FLOWERING 6 (REF6), directly promotes the expression of its ten target senescence regulatory and functional genes (*EIN2*, *ORE1*, *NAP*, *AtNAC3*, *NTL9*, *NYE1*/*2*, *LOX1*, *PAD4*, and *PPDK*), which are involved in major phytohormones’ signaling, biosynthesis, and chlorophyll degradation. Crucially, REF6 is substantially involved in promoting the H3K27me3 demethylation of both their promoter and/or coding regions during the aging process of leaves. We therefore provide conclusive evidence that H3K27me3 methylation is an epigenetic mechanism hindering the premature transcriptional activation of key *SAGs*, which helps to explain the “aging effect” of senescence initiation.

## Introduction

Histone methylation plays an essential role in diverse biological processes, ranging from transcriptional regulation to heterochromatin formation. Histone lysine methyltransferases (“writers”) and demethylases (“eraser”) dynamically regulate methylation levels, and in *Arabidopsis*, methylations at Lys4 (K4), Lys9 (K9), Lys27 (K27), and Lys36 (K36) of histone H3 have been extensively studied [1]. In general, histone H3K9 and H3K27 methylation are associated with silenced regions, whereas H3K4 and H3K36 methylation with active genes [2]. H3K9me1/2 and H3K27me1 are enriched at constitutively silenced heterochromatin in *Arabidopsis* [3, 4]. H3K27me3 repression of gene expression during development is a conserved mechanism in eukaryotes, and several thousand genes (more than 15% of all transcribed genes) in *Arabidopsis* are marked by the modification [5–7]. H3K27me3 is catalyzed by polycomb repressive complex 2 (PRC2), which consists of four parts: E(Z), Su(z)12, p55, and Esc. Sequence similarity and genetic analyses revealed that *Arabidopsis* EZH2 homologs, including curly leaf (CLF), swinger (SWN), and medea (MEA), are H3K27me3 methyltransferases [1, 5].

The Jumonji (JMJ) protein was first identified in mouse by a gene trap approach [8]. Then, JMJ domain-containing proteins were found to be able to remove histone methyl groups [9, 10]. *Arabidopsis* JMJ homologs, RELATIVE OF EARLY FLOWERING 6 (REF6), EARLY FLOWERING 6 (ELF6) and JMJ30, were demonstrated as H3K27me3 demethylases [11–13]. ELF6 was first reported as a repressor of flowering in the photoperiod pathway, and REF6, with the highest similarity to ELF6, as a flowering locus C (FLC) repressor [14]. Loss-of-function mutations in REF6 lead to the ectopic accumulation of H3K27me3 at hundreds of genes in seedlings, suggesting that REF6 is a coordinator of multiple developmental programs in plants [11]. BRI1-EMS-suppressor 1 (BES1) and Nuclear transcription factor Y (NF-YA) were reported to recruit REF6 to its target genes [15, 16]. Notably, a new targeting mechanism of REF6 was recently revealed, i.e. by directly binding the CTCTGYTY motif in its target genes [17, 18]. In senescing leaves, the reprogrammed distribution of H3K4me3 and H3K27me3 accompanies a decondensation of chromocenter heterochromatin in the interphase nuclei [19]. A further report showed that the senescence-associated global changes at chromatin organization can be inhibited by overexpressing *SUVH2*, a gene encoding a methyltransferase [20]. The above reports reveal a close relationship between senescence and histone modification, but how histone modification precisely regulates leaf senescence remains unclear.

Plants senesce typically in the modular manner and leaves are the major modular organ. Leaf senescence is an integral part of plant development, triggering characteristic degenerative processes as an adaptive mechanism, such as chlorophyll (Chl) degradation and macromolecule breakdown, and particularly recycling of released nutrients to nascent tissues or storage organs [21–23]. De-greening, reflecting a net loss of Chl, is the most obvious symptom of leaf senescence, which is closely associated with the degradation of light-harvesting complexes (LHCs) [24, 25]. The biochemical pathway of de-greening has been largely elucidated by the identification of *Chl catabolic genes* (*CCGs*) or Chl catabolic enzymes (CCEs) in *Arabidopsis* as well as in rice [26]. During senescence, Chl *b* is converted to Chl *a* via the catalysis of Chl *b* reductase (encoded by NYC1/NOL) and 7-hydroxymethyl Chl *a* reductase (HCAR) [27–30]. NYEs/SGRs-catalyzed magnesium dechelation is the first step of Chl *a* degradation, generating pheophytin *a* [31], which is then sequentially degraded/modified to: 1) pheophorbide *a* by pheophytin pheophorbide hydrolase (PPH) [32, 33], 2) to red chlorophyll catabolites (RCC) by pheophorbide a oxygenase (PAO), 3) to primary fluorescent chlorophyll catabolites (*p*FCC) by red chlorophyll catabolite reductase (RCCR) [34], and 4) finally to hydroxy-*p*FCC by TIC55 inside the chloroplast [35]. The conversion from pheophorbide *a* to RCC leads to the loss of green color of Chl catabolites [34]. The identification of NYE1/SGR1, among other major CCEs, was an important event, not only because it is responsible for catalyzing the first step of Chl *a* degradation but more significantly because its mutation is responsible for Mendel’s green cotyledon trait [36–43].

A broad range of endogenous factors as well as environmental cues can modulate the initiation/progression of leaf senescence [22], and ethylene (ET) has been shown to be a key promoter [44–47]. EIN2 and EIN3 predominantly mediate its signaling via a sophisticated regulatory hierarchy [46, 48–51]. During leaf senescence, EIN3 directly activates the expression of *ORE1*/*NAC2* and *NAP*, as well as *CCGs*, to accelerate leaf senescence [52]. EIN3 is also involved in a feed-forward regulation by directly suppressing the expression of *miR164*, which targets *ORE1* at the post-transcriptional level [46, 49]. Moreover, ORE1 and NAP directly activate the expression of *Senescence associated genes* (*SAGs*) and *CCGs* to accelerate leaf Chl degradation and senescence in general [52–54].

It is important to note that ethylene can promote senescence only in the leaves that have reached a certain age, in which some age-related changes must have occurred [55, 56]. Yet, the identities of these changes have not been clearly defined, and particularly, the mechanism(s) by which the transcription of the genes encoding major senescence regulatory as well as functional *SAGs*/*CCGs* is prematurely repressed remains elusive. In this study, we employed the yeast one-hybrid (Y1H) system to screen for the putative transcriptional regulator of *NYE1* and interestingly, identified REF6, a histone H3 lysine 27 demethylase, as a candidate. It was then confirmed that REF6 modulates Chl degradation by directly up-regulating the transcription of *NYE1*/*2*. Subsequently, we demonstrated that REF6 also regulates general leaf senescence, and identified other eight senescence regulatory and functional genes (*EIN2*, *ORE1*, *NAP*, *PPDK*, *PAD4*, *LOX1*, *AtNAC3*, and *NTL9*) as its direct targets. Finally, we showed that REF6 regulates the expression of its ten target *SAGs* by reducing their H3K27me3 levels. Our study identifies that H3K27me3 methylation represents a kind of epigenetic mechanisms prohibiting the premature activation of leaf senescence in *Arabidopsis*.

## Results

### REF6 directly binds to both the promoters and coding regions of *NYE1* and *NYE2*

**Fig 1:**
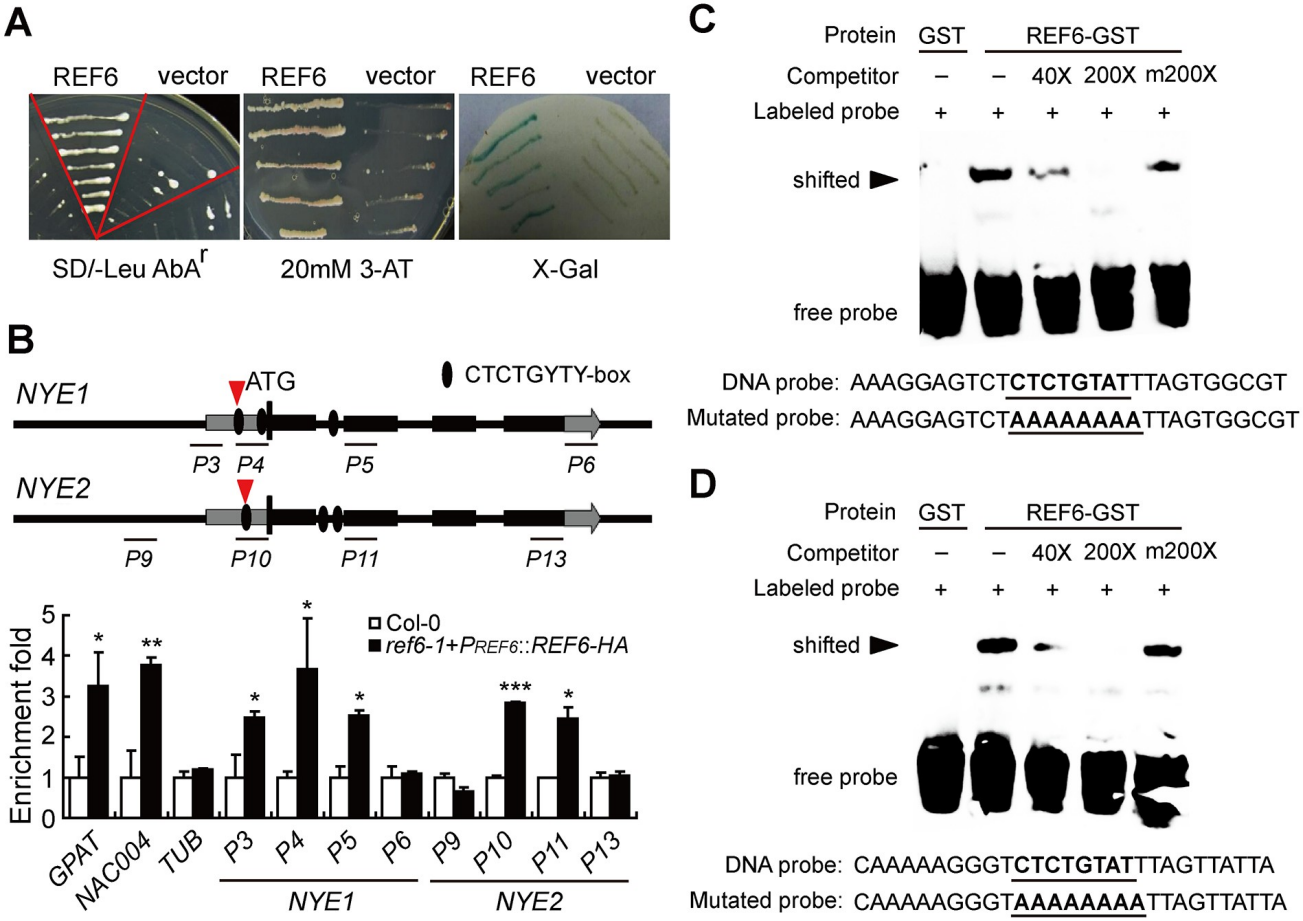
REF6 directly binds to both the promoters and coding regions of *NYE1* and *NYE2*. (A) Physical interactions of REF6 with *NYE1* promoter in Y1H assays and blue-white spotting tests. (B) ChIP assays of *in vivo* association of REF6-HA with *NYE1/2* genes in the 10-day-old seedlings of *ref6-1*+*PREF6::REF6-HA* and Col-0. Fold enrichments were calculated as the ratios of the signals in *ref6-1*+P*REF6::REF6-HA* to the signals in Col-0. Primers are listed in S4 Table. *GPAT4* and *NAC004* genes were used as positive controls, with *TUB* as a negative control. Data are mean ± SD (n = 3). *P < 0.05, **P < 0.01, ***P < 0.001 by paired Student’s t test. (C, D) EMSAs of *in vitro* binding of REF6 to the CTCTGYTY motifs within the ChIP-PCR fragments P4 and P10 of *NYE1* (C) and *NYE2* (D) promoters (indicated with red arrows). Probe sequences used in EMSA are shown in (C) and (D). GST-tagged REF6 was incubated with the biotin-labeled wild-type DNA probe. Competition experiments were performed by adding the excessive amounts (40× and 200×, respectively) of unlabeled DNA probe. A mutated probe was used to test binding specificity. Shifted bands, indicating the formation of DNA-protein complexes, are indicated by arrows. “-” represents absence, “+” represents presence. Sequences of both the wild-type and mutated probes are shown on the bottom of the images.

NYE1 was initially identified as a crucial regulator of Chl degradation during green organ senescence in diversified species and particularly shown to be responsible for the green/yellow cotyledon trait of Mendel’s pea (*Pisum sativum*) [36–43]. To understand the transcriptional regulation of *NYE1*, we exploited Y1H to screen for the putative trans-regulators of *NYE1*. The core part of *NYE1*’s promoter (-532 bp upstream of its ATG) was used as the bait for the screening against a cDNA library generated from the senescing leaves of *Arabidopsis* plants [57]. To our surprise, a positive clone encoding a zinc finger structure protein with two JMJ domains was identified, which was previously demonstrated to be a histone H3K27me3 demethylase and named as REF6 (AT3G48430) [11]. We then cloned the full-length coding region of *REF6* into the vector p*GAD-T7* and introduced the resultant construct along with *P*_*NYE1*_::*Pabai* into Y1H Gold. Y1H Gold grew well on a medium containing *Aureobasidin A*. To confirm this result, we generated a construct containing the reporter genes *HIS3* and *LacZ* driven by the *NYE1*’s promoter and introduced it into the yeast YM4271 along with empty p*GAD-T7* or *REF6*::p*GAD-T7*. Compared with the yeast transformed with empty p*GAD-T7*, the one with *REF6*::p*GAD-T7* grew well in a 3-AT-containing medium and turned blue when transferred to X-Gal-containing medium (Fig 1A).

Remarkably, REF6’s targeting mechanism was recently revealed, i.e. REF6 directly binds to the CTCTGYTY motif of its target genes [17]. By scanning *NYE1*’s promoter and coding regions, we found three CTCTGYTY motifs, and interestingly, we also detected three CTCTGYTY motifs across the promoter and coding regions of *NYE2*, a functional paralog of *NYE1* [58]. To test whether REF6 protein could directly bind to these motifs *in vivo*, we carried out chromatin immuneprecipitation (ChIP)-qPCR assays with multiple pairs of primers designed accordingly. Chromatins isolated from *ref6-1*+*P*_*REF6*_::*REF6-HA* transgenic plants were immuneprecipitated with HA antibody, and RT-qPCR was then performed to quantify the enrichment of corresponding promoter and coding regions. We observed 2.6- to 3.7-fold enrichments in the *NYE1*’s promoter region (*NYE1*-P3, -P4), where the first two CTCTGYTY motifs are located, and relatively less enrichment in the first exon (*NYE1*-P5), which is close to the third CTCTGYTY motif, in contrast to no enrichment at the end of the coding region (*NYE1*-P6), where no CTCTGYTY motifs were detected. An expected enrichment pattern was also observed within *NYE2*’s promoter and coding regions (Fig 1B).

Electrophoretic mobility shift assay (EMSA) was carried out to determine whether REF6 protein could directly bind to *NYE1*/*2* promoters *in vitro*. The C2H2-ZnF domain of REF6 fused with a GST tag (GST-REF6C 1,239–1,360 aa) was expressed and purified as described previously [17]; and a 28 bp DNA fragment covering the first CTCTGYTY motif in *NYE1* promoter was made a probe. We detected a shifted band when labeled probes were pre-incubated with GST-REF6C, and addition of excess unlabeled probes competed with the binding. In contrast, REF6 protein did not bind to the motif-mutated probes (Fig 1C), indicating that REF6 specifically bound to the motif of *NYE1* promoter *in vitro*. REF6 protein could also specifically bind to the motif of *NYE2* promoter *in vitro* (Fig 1D). These observations, along with the previous data, collectively suggest that REF6 may act as a transcriptional regulator of *NYE1*/*2* by directly binding to their CTCTGYTY motif-containing regions.

### REF6 promotes Chl degradation during leaf senescence via up-regulation of *NYE1* and *NYE2*

**Fig 2:**
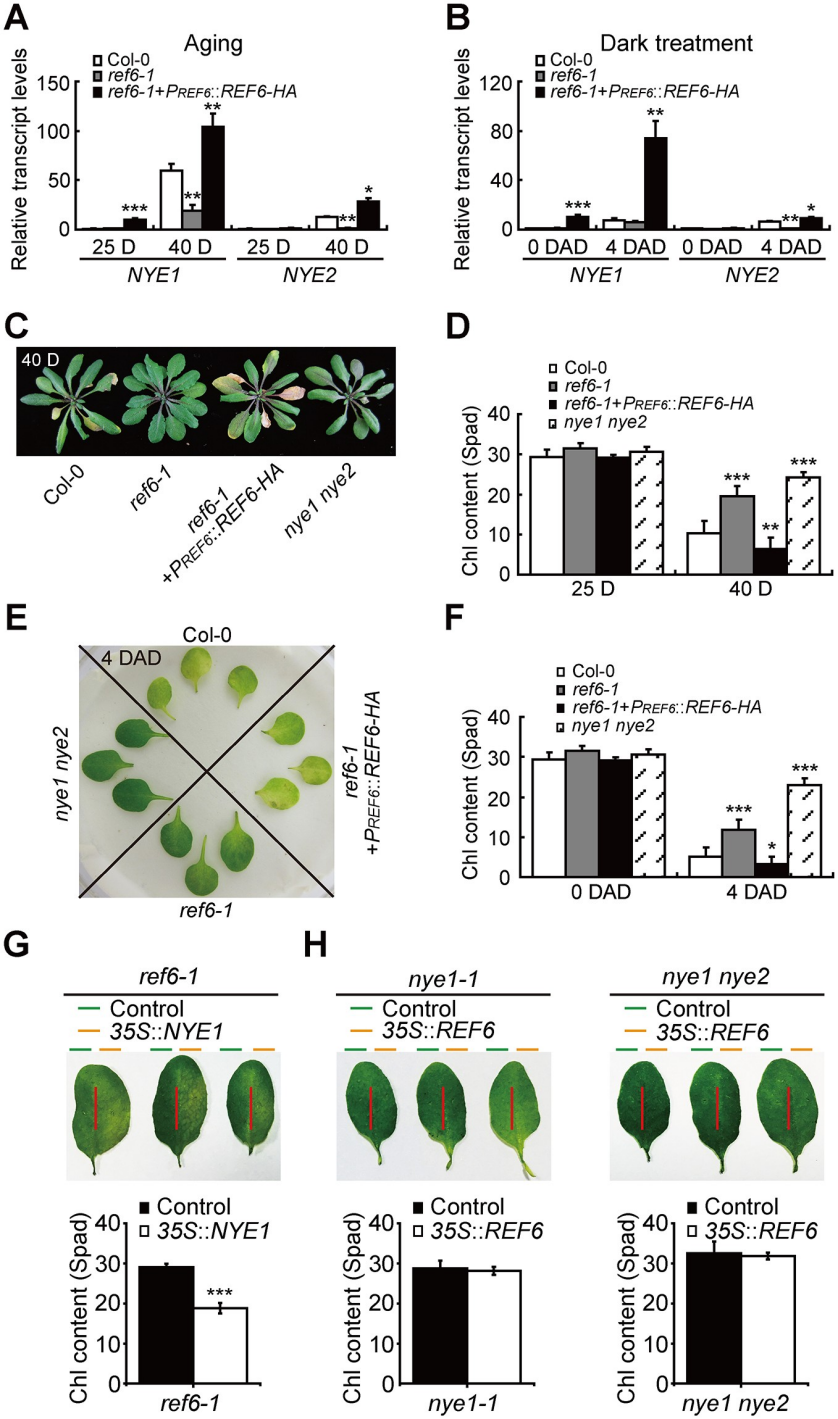
REF6 promotes both Chl degradation and general leaf senescence process. (A, B) Changes in the transcript levels of *NYE1/2* in *ref6-1* and *ref6-1+PREF6::REF6-HA* relative to those in Col-0 with aging (A) and during dark treatment (B) (DAD, days after dark treatment). Data are mean ± SD (n = 3). *P < 0.05, **P < 0.01, ***P < 0.001 by paired Student’s t test. (C, D) Plants of indicated genotypes (*ref6-1*, r*ef6-1+PREF6::REF6-HA*, *nye1 nye2* as well as Col-0) grown under long day-growth conditions for 40 days (C). Chl contents (D) in the leaves of the plants shown in (C). (E, F) Phenotypes of indicated genotypes on 4 DAD. Detached leaves were obtained from 25-day-old plants under long day-growth conditions. Unless stated otherwise, the 3rd and 4th detached rosette leaves were used for physiological and molecular analyses. (G) Leaf phenotypes and Chl contents of *ref6-1* after infiltration with Agrobacterium containing 35S::NYE1 or empty vector pCHF3. (H) Leaf phenotypes and Chl contents of *nye1-1* or *nye1 nye2* after infiltration with Agrobacterium containing 35S::REF6 or empty vector pCHF3.

To verify the above assumption, we examined the characteristic changes of *NYE1*/*2*’s transcriptions in *ref6-1* and *ref6-1*+*P*_*REF6*_::*REF6-HA*, as well as in Col-0, during age-triggered and dark-induced leaf senescence. The *ref6-1*+*P*_*REF6*_::*REF6-HA* were the native promoter-driven *REF6* overexpression plants with ~3.0-fold enhancement in its transcription (S1 Fig). Loss-of-function of REF6 significantly reduced the transcription of *NYE1*/*2*, whereas overexpression of *REF6* enhanced the transcription of *NYE1*/*2* during both of the scenarios of leaf senescence (Fig 2A and 2B).

To examine the role of REF6 in regulating Chl degradation, we first characterized the de-greening phenotype of *ref6-1* and *ref6-1*+*P*_*REF6*_::*REF6-HA* during age-triggered senescence, with *nye1 nye2* and Col-0 plants used as controls. As expected, the rosette leaves of *ref6-1* showed an obvious stay-green phenotype as compared with Col-0, whereas *ref6-1*+*P*_*REF6*_::*REF6-HA* exhibited a premature yellowing phenotype, in contrast to the most severe stay-green phenotype of *nye1 nye2* (Fig 2C). Measurements of Chl content were consistent with the visual phenotype (Fig 2D). To confirm the regulatory role of REF6 in Chl degradation, their 3rd and 4th rosette leaves were incubated in darkness and characterized four days after dark treatment (DAD). A stay-green phenotype was also observed on the leaves of *ref6-1*, in contrast to an obvious premature yellowing on those of *ref6-1*+*P*_*REF6*_::*REF6-HA* (Fig 2E). Their phenotypic observations were validated by their measurements of Chl content (Fig 2F).

Finally, we checked whether NYE1 overexpression could rescue the stay-green phenotype of *ref6-1* by using an *in situ* transient expression system. We first infiltrated one half of a *ref6-1*’s leaf with *Agrobacteria* containing *NYE1* expression vector and found that it turned yellow two days after infiltration while the other half of the leaf transfected with *Agrobacteria* containing the empty plasmid still stayed green (Fig 2G). Then, we overexpressed *REF6* in *nye1-1* and *nye1 nye2* in the same manner and detected similar stay-green phenotypes between the two halves of a leaf transfected (Fig 2H). All the analyses convincingly demonstrate that REF6 requires *NYE1*/*2* for promotion of Chl degradation during leaf senescence.

### REF6 positively regulates the general leaf senescence process

**Fig 3:**
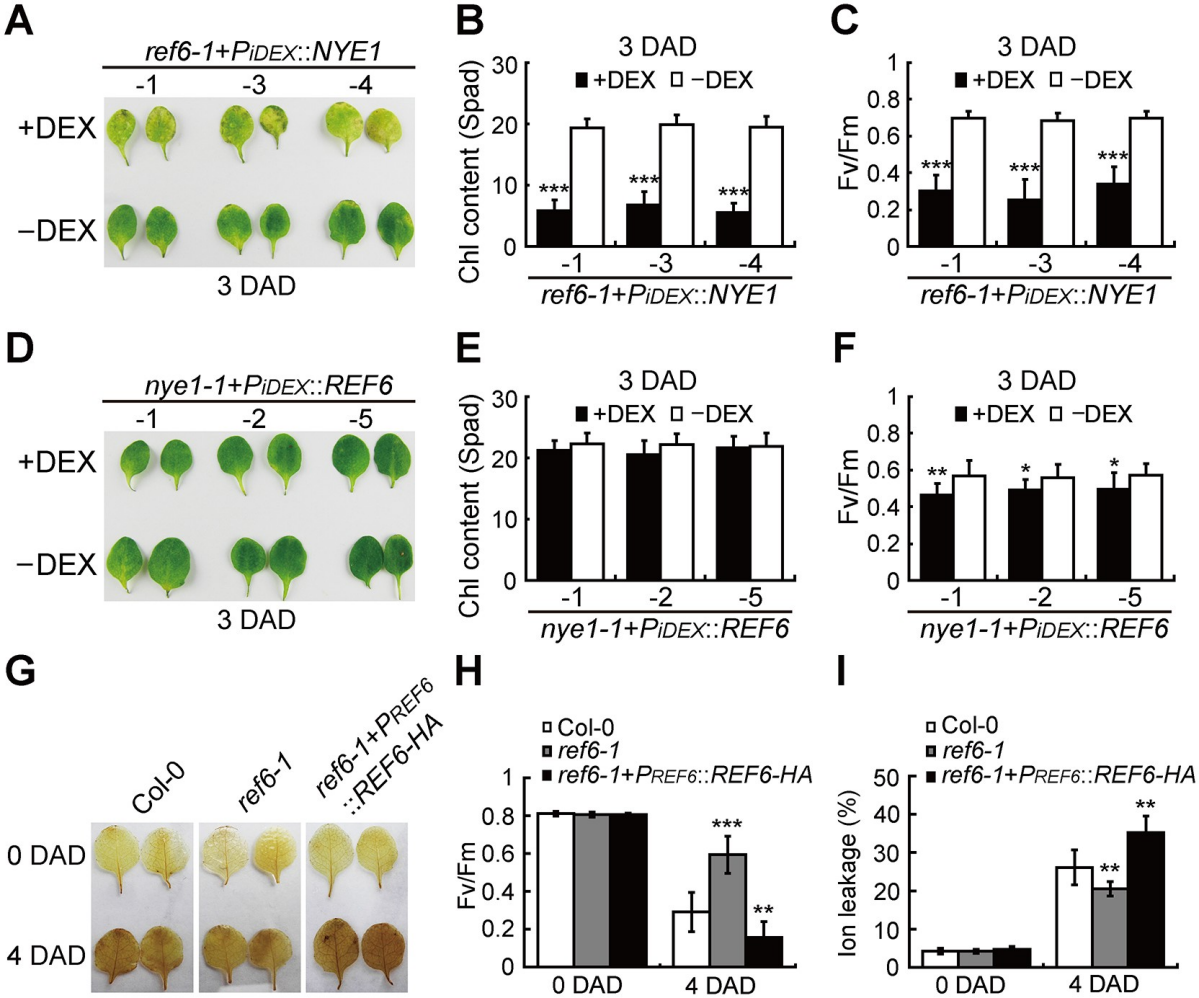
Overexpression of *REF6* and *NYE1* in each other’s mutants confirms their roles in promoting the general leaf senescence process. (A) Leaf phenotypes of *ref6-1+PiDEX::NYE1* transgenic plants treated with or without DEX on 3 DAD. -1, -3, and -4 represent different transgenic lines. (B, C) Chl contents (B) and Fv/Fm ratios (C) in the leaves shown in (A). (D) Leaf phenotypes of *nye1-1+PiDEX::REF6* transgenic plants treated with or without DEX on 3 DAD. -1, -2, and -5 represent different transgenic lines. (E, F) Chl contents (E) and Fv/Fm ratios (F) in the leaves shown in (D). (G-I) H*2*O*2* levels detected by DAB staining (G), Fv/Fm ratios (H), and Ion leakage (I), in ref6-1, Col-0, and *ref6-1+PREF6::REF6-HA* plants on 4 DAD. Data are mean ± SD (n = 10). *P < 0.05, **P < 0.01, ***P < 0.001 by unpaired Student’s t test.

To confirm the above transient expression results, we overexpressed *NYE1* in *ref6-1* plants (*ref6-1*+*P*_*iDEX*_::*NYE1*) and *vise versa*, *REF6* in *nye1-1* plants (*nye1-1*+*P*_*iDEX*_::*REF6*) by using a dexamethasone (DEX) induction system. After treated with 30 μM chemical inducer DEX, *ref6-1*+*P*_*iDEX*_::*NYE1* transgenic plants exhibited a yellowing phenotype compared with the untreated control on 3 DAD (Fig 3A and 3B and S2A Fig), whereas *nye1-1*+*P*_*iDEX*_::*REF6* transgenic plants displayed a stay-green phenotype similar to that of *nye1-1* (Fig 3D and 3E and S2B Fig). These findings are consistent with the transient expression results. Interestingly, although Chl degradation was not affected, a significantly decreased Fv/Fm ratio was detected in *nye1-1*+*P*_*iDEX*_::*REF6* transgenic plants compared with un-induced controls (Fig 3C and 3F), which reminded us of that REF6 might also be involved in the regulation of other *SAGs* apart from *NYEs*.

Subsequently, we examined the major senescence parameters of both *REF6*’s loss-of-function mutant (*ref6-1*) and its native promoter-driven overexpression lines (*ref6-1*+*P*_*REF6*_::*REF6-HA*) during both dark-induced and age-triggered leaf senescence. It was found that, on 4 DAD, the Fv/Fm ratio remained higher while the ion leakage and H_2_O_2_ content lower in the 3rd and 4th detached rosette leaves of 25-day-old *ref6-1* plants than those in Col-0, and the exact opposite trends in the changes of the senescence parameters were observed in *ref6-1*+*P*_*REF6*_::*REF6-HA* (Fig 3G–3I) [59, 60]. Consistently, 40 days after germination under long-day growth conditions, a significantly higher Fv/Fm ratio and a significantly lower Fv/Fm ratio were detected in the 3rd and 4th rosette leaves of *ref6-1* and *ref6-1*+*P*_*REF6*_::*REF6-HA* plants, respectively (S3 Fig). These results confirm that REF6 indeed regulates the general leaf senescence process.

### REF6 promotes the transcription of dozens of *SAGs* during leaf senescence

**Fig 4:**
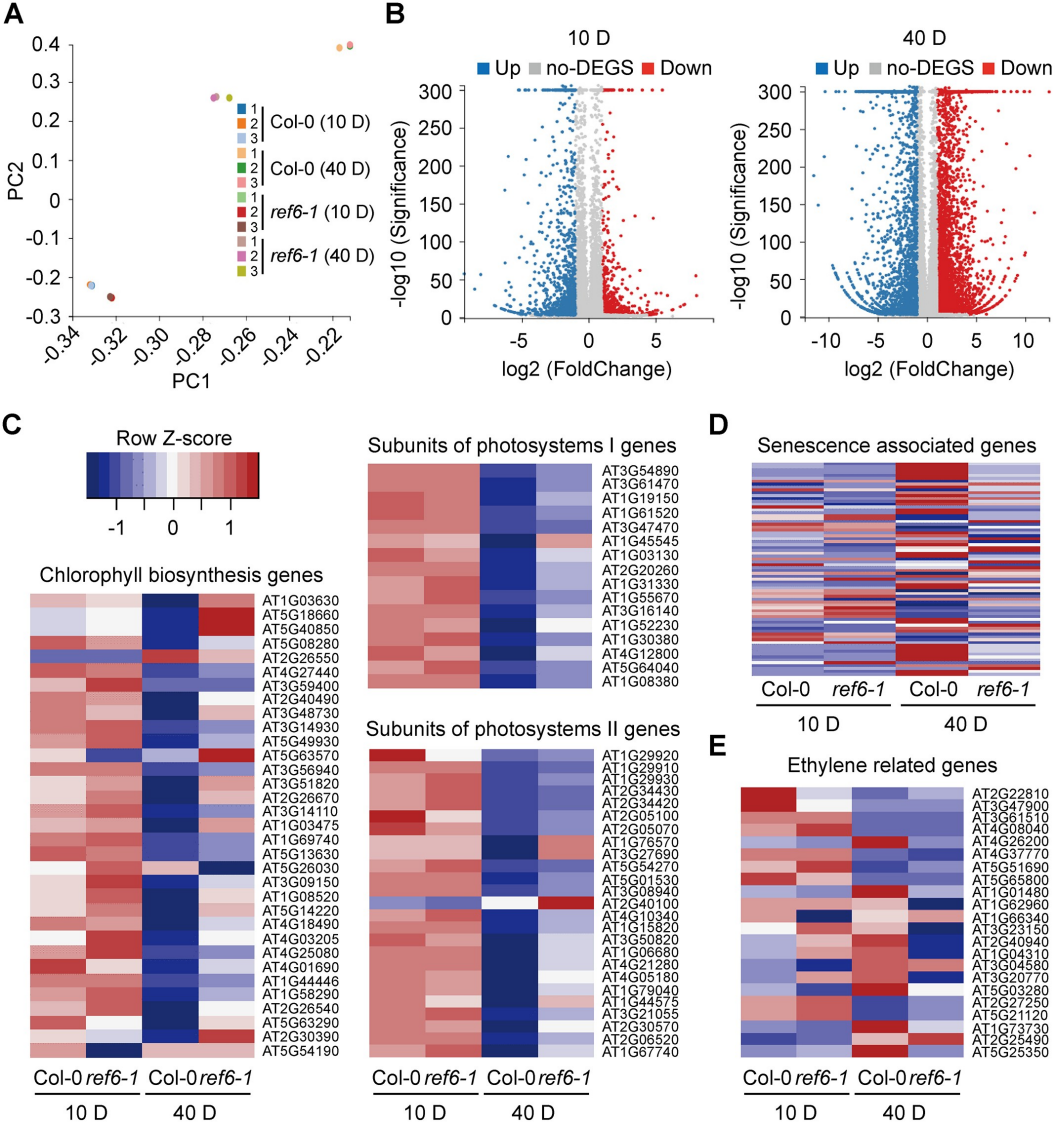
Transcriptome analysis of Col-0 and *ref6-1* mutant. (A) Principle component analysis of log2-transformed transcriptome data of 12 samples. (B) Volcano plot of the differentially expressed genes (DEGs) for Col-0 and *ref6-1* investigated in this work (10-day-old seedlings, left; 40-day-old rosette leaves, right). The y-axis corresponds to the mean expression value of -log10 (q-value), and the x- axis displays the log2-fold change value. (C-E) Heat map showing the expression of chlorophyll biosynthesis, subunit genes of photosystems I and II (C), senescence associated genes (D), and ethylene related genes (E) in 10-day-seedlings and 40-day-old leaves of Col-0 and *ref6-1*.

To examine the effect of loss-of-REF6 function on the global gene expression pattern, we did a comparative RNA-seq analysis of the 10-day-old seedlings and 40-day-old rosette leaves of both *ref6-1* and Col-0 plants. In total, 25,044 expressed genes were identified from all the samples. PCA analysis showed that in the rosette leaves, the transcriptional difference between Col-0 and *ref6-1* was much more significant in comparison to that in the seedlings (Fig 4A): a total of 6,106 differentially expressed genes (DEGs, those with more than 2.0-fold change in transcription) were identified in the rosette leaves compared to 1,319 DEGs in the seedlings (Fig 4B and S1 Table). The decline in leaf photosynthetic capacity is correlated with the progression of leaf senescence [61], and expectedly, a large number of Chlorophyll biosynthesis- and photosynthesis-related genes were down-regulated much less rapidly in *ref6-1* than those in Col-0 (Fig 4C and S2 Table). By contrast, among the previously identified 74 *SAGs* [62], 33 were up-regulated far less significantly in *ref6-1* than those in Col-0 (Fig 4D and S2 Table). These analyses provide evidence that REF6 significantly promotes the general leaf senescence process via up-regulating the transcription of major *SAGs*. Notably, by referring to the *SAGs* database set up by Liu et al. [62], we found that the up-regulated transcription of quite a few of the *SAGs* relating to ethylene biosynthesis, signaling or response was significantly compromised in the 40-day-old rosette leaves of *ref6-1* (Fig 4E and S2 Table), implying that ethylene signaling and/or biosynthesis might be largely responsible for mediating REF6-regulated leaf senescence.

### REF6 directly activates the transcription of its target *SAGs*

**Fig 5:**
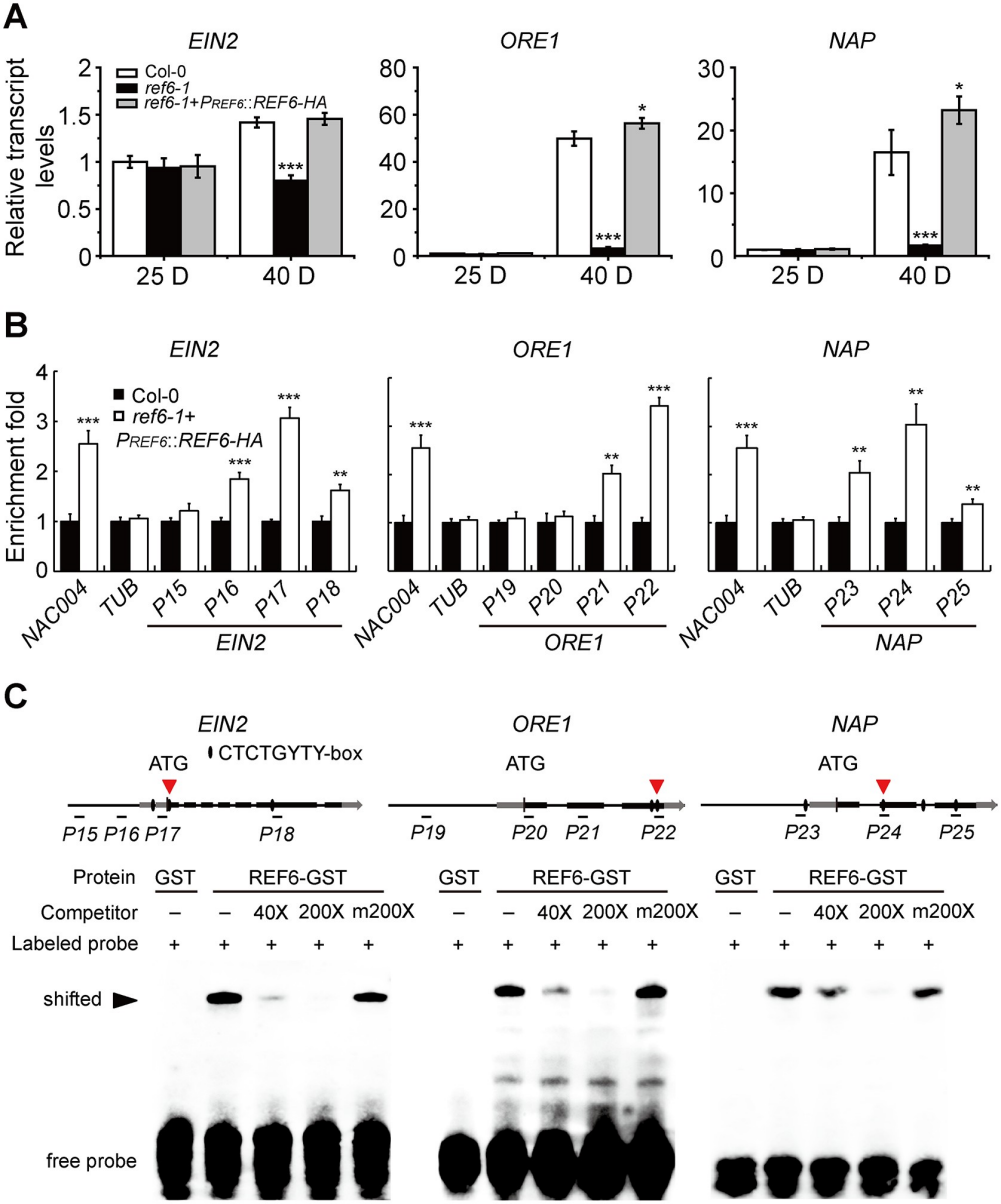
REF6 directly up-regulates the transcription of *EIN2, ORE1,* and *NAP*. (A) Relative transcript levels of *EIN2, ORE1*, and *NAP* genes in the leaves detached from 25-day-old or 40-day-old Col-0, *ref6-1*, and *ref6-1+PREF6::REF6-HA* plants grown under long day-growth conditions. (B) ChIP assays of *in vivo* binding of REF6-HA to *EIN2*, *ORE1*, and *NAP* promoters and coding regions in the 10-day-old seedlings of *ref6-1*+*PREF6::REF6-HA* and Col-0 plants. Fold enrichments were calculated as described previously. Primers are listed in S4 Table. *NAC004* gene was used as a positive control, whereas *TUB* as a negative control. In (A) and (B), data are mean ± SD (n = 3). *P < 0.05, **P < 0.01, ***P < 0.001 by paired Student’s t test. (C) EMSAs of *in vitro* binding of REF6 to the CTCTGYTY motifs within the ChIP-PCR fragments P17, P22, and P24 of *EIN2*, *ORE1*, and *NAP* coding regions (indicated with red arrows). Probe sequences used in EMSA are: 5’-AGGAACCATTCTCTGGATAAACCCTAGC-3’ for *EIN2*, 5’-TACTCGGATCCTCTGTTTTTACAAGACA-3’ for *ORE1*, and 5’-TTTCTCCAAACTCTGTTTTCTCTGTAAA-3’ for *NAP*.

To identify the *SAGs* directly activated by REF6, we analyzed the overlap between REF6 target genes [11] and the *SAGs* significantly down-regulated in the 40-day-old rosette leaves of *ref6-1* (S3 Table), and revealed three ethylene signaling genes, *EIN2*, *ORE1*, and *NAP* [51, 63], as well as other five *SAGs*, *PPDK*, *PAD4*, *LOX1*, *AtNAC3*, and *NTL9*, as REF6 candidate target *SAGs*. The ethylene signaling pathway has been elucidated as a key hormonal signaling pathway in regulating age-triggered leaf senescence [49, 51, 52], and we therefore focused on the analysis of *EIN2*, *ORE1*, and *NAP*’s involvement. To preliminarily examine the regulatory relationship of the three ethylene signaling genes with REF6, we measured their transcript levels and found that their enhancements in transcription with aging were significantly reduced in *ref6-1* while apparently increased in *ref6-1*+*P*_*REF6*_::*REF6-HA* compared to those in Col-0 (Fig 5A). The *in vivo* association of REF6 with the three genes was examined by ChIP-qPCR assays, and it was found that REF6 was associated with all the three genes in their coding regions but not in their promoter regions, with 3.0-, 3.5-, and 3.0-fold enrichments in the coding regions of *EIN2* (*EIN2*-*P17*), *ORE1* (*ORE1*-*P22*), and *NAP* (*NAP*-*P24*), respectively (Fig 5B). By scanning their genomic regions, CTCTGYTY motifs were identified mainly in their coding regions instead of their promoters. With the ChIP data as a reference, an EMSA was performed to examine whether REF6 protein could directly bind to the CTCTGYTY motifs in their coding regions. Indeed, bindings were detected within the coding regions of all the three genes (Fig 5C). These results indicate that REF6 modulates leaf senescence likely by directly up-regulating *EIN2*, *ORE1*, and *NAP*, and presumably, other five candidate target *SAGs* as well (CTCTGYTY motifs were also detected in their coding/promoter regions (S4A Fig).

### REF6 promotes the transcription of its ten target *SAGs* by reducing their H3K27me3 levels

**Fig 6:**
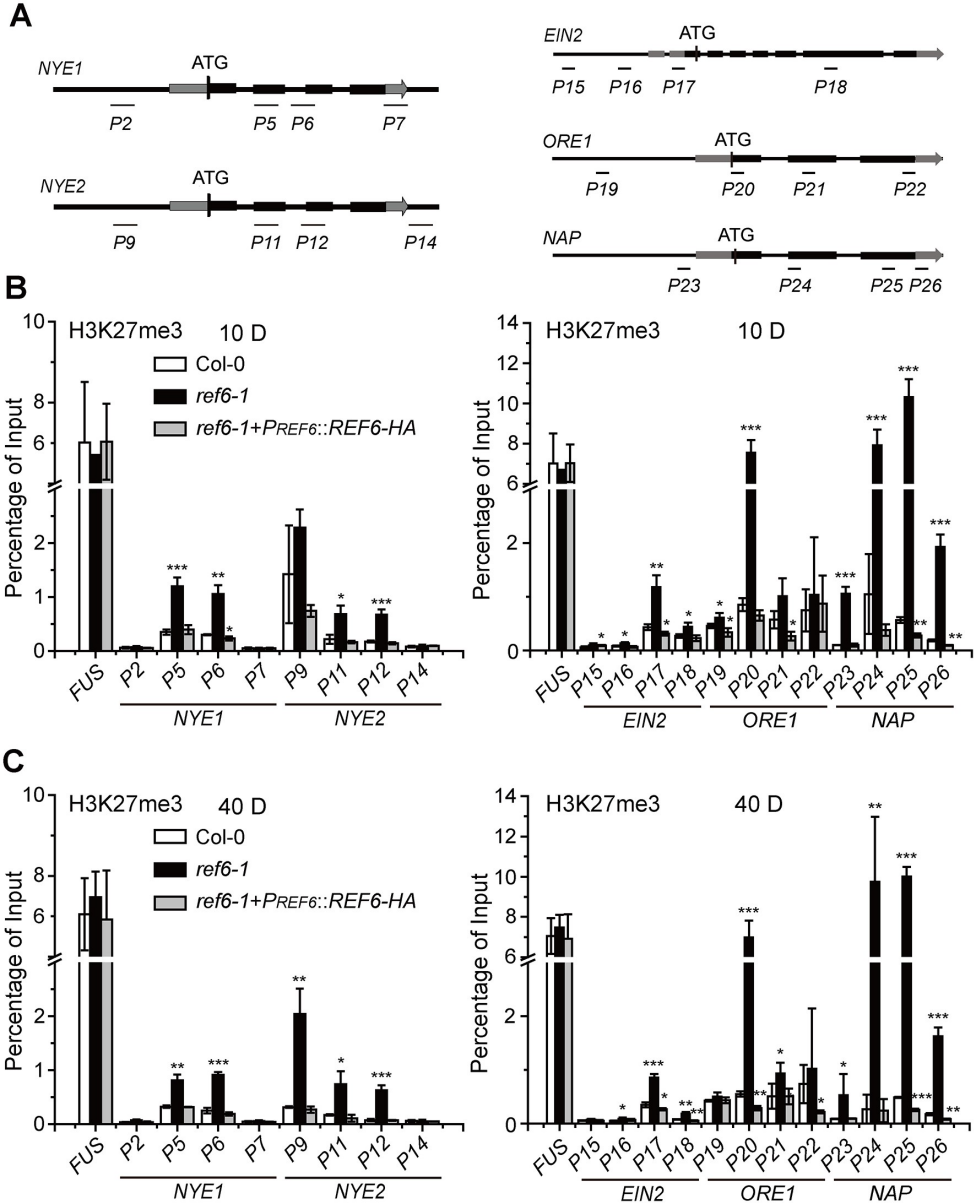
Loss-of-function of REF6 increases H3K27me3 levels at *NYE1/2*, *EIN2*, *ORE1*, and *NAP* genes. (A) Schematic diagrams of *NYE1/2*, *EIN2*, *ORE1*, and *NAP* genes and positions of the primers used for determining their H3K27me3 levels. Primer sequences are listed in S4 Table. (B) H3K27me3 levels of *NYE1/2*, *EIN2*, *ORE1*, and *NAP* genes, expressed as the percentage of input, in the 10-day-old seedlings of Col-0, *ref6-1*, and *ref6-1+PREF6::REF6-HA* grown under long day-growth conditions. (C) H3K27me3 levels of *NYE1/2*, *EIN2*, *ORE1*, and *NAP* genes in the leaves detached from the 40-day-old plants of Col-0, *ref6-1*, and *ref6-1+PREF6::REF6-HA* grown under long day-growth conditions. In (B)—(C), data are mean ± SD (n = 3). *P < 0.05, **P < 0.01, ***P < 0.001 by paired Student’s t test.

Previous reports revealed that the expression of genes is tightly restricted by a high level of H3K27me3 [6], and loss-of-function of REF6 caused a genome-wide H3K27me3 hypermethylation [11]. We then hypothesized that REF6 might directly promote the transcription of its target *SAGs* by reducing their H3K27me3 levels. To test this hypothesis, we measured H3K27me3 level at these *SAGs* in the 10-day-old seedlings (Fig 6A and 6B and S4A and S4B Fig) and 40-day-old leaves (Fig 6A and 6C and S4A and S4C Fig) of both *ref6-1* and Col-0 plants. It was detected that H3K27me3 levels were significantly higher in *ref6-1* than in Col-0 plants at all the ten *SAGs*, and notably, between *ref6-1* and Col-0, much bigger differences at H3K27me3 level were revealed at *ORE1*, *NAP*, *NAC3*, and *NTL9* in both the 10-day-old seedlings and the 40-day-old leaves (Fig 6 and S4 Fig). We also found that H3K27me3 levels at all the ten genes were lower in the 40-day-old leaves than those in the 10-day-old seedlings of both Col-0 and *ref6-1* plants, suggesting that the H3K27me3 on these *SAGs* are gradually cleared up by REF6 as well as other related demethylases towards the initiation of leaf senescence. These data demonstrate that REF6 facilitates the expression of its ten target *SAGs* during the initiation/progression of leaf senescence by reducing their H3K27me3 levels.

### REF6-promoted leaf senescence is independent of plant development

**Fig 7:**
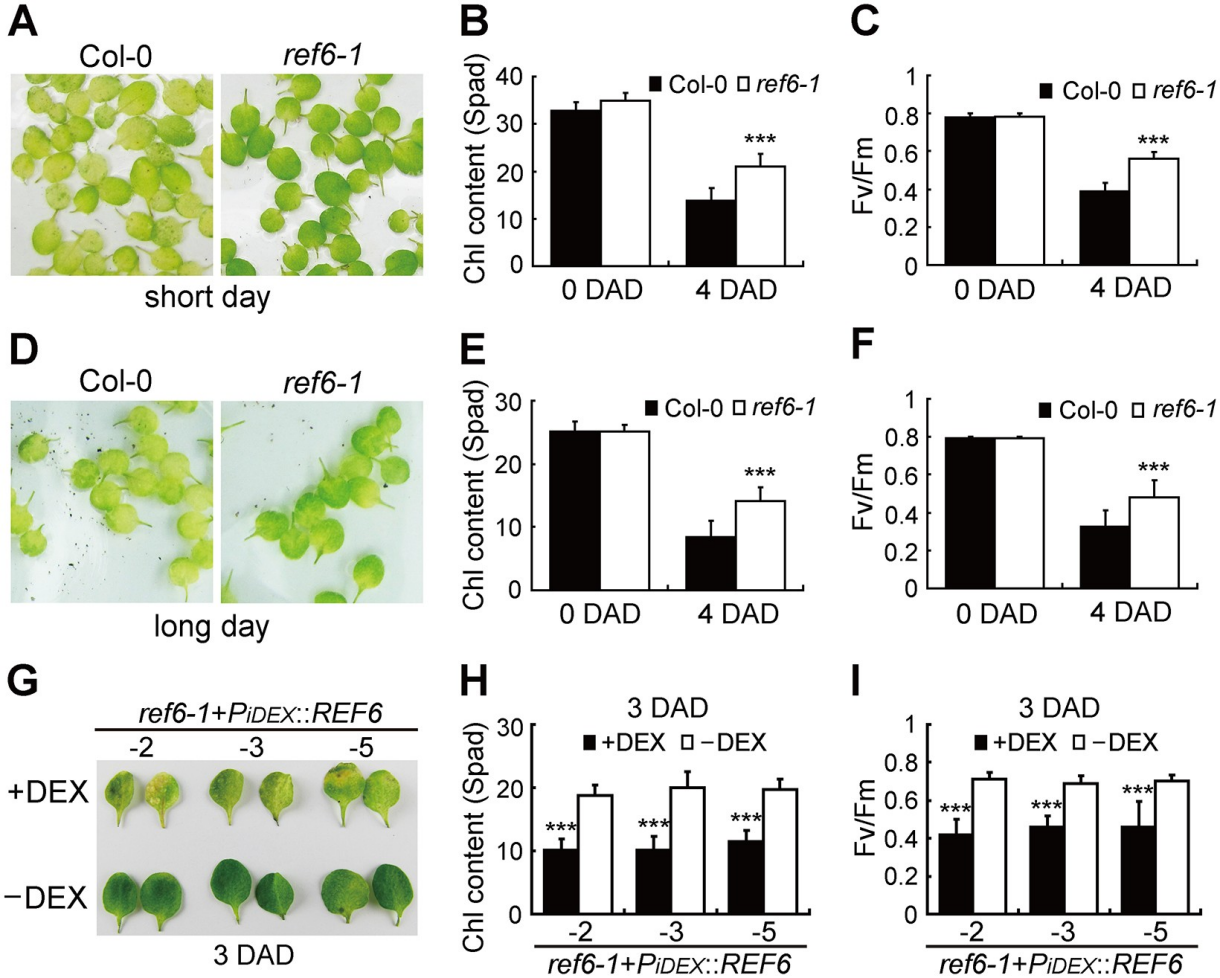
REF6 regulates leaf senescence independently of plant development. (A) Phenotypes of the leaves detached from 40-day-old *ref6-1* and Col-0 plants grown under short day-growth conditions on 4 DAD. (B, C) Chl contents (B) and Fv/Fm ratios (C) in the leaves shown in (A). (D) Phenotypes of the leaves detached from 14-day-old *ref6-1* and Col-0 plants grown under long day-growth conditions on 4 DAD. (E, F) Chl contents (E) and Fv/Fm ratios (F) in the leaves shown in (D). (G) Leaf phenotypes of *ref6-1*+*PiDEX::REF6* transgenic plants treated with or without DEX on 3 DAD. -2, -3, and -5 represent different transgenic lines. (H, I) Chl contents (H) and Fv/Fm ratios (I) in the leaves shown in (G). In (B), (C), (E), (F), (H), and (I), data are mean ± SD (n = 10). ***P < 0.001 by unpaired Student’s t test.

It has been found that early leaf senescence is always accompanied with early flowering, but delayed leaf senescence does not necessarily cause late flowering [64]. Some histone modification factors were reported to affect both flowering time and leaf senescence process [19, 20, 24]. Since *ref6-1* is an obvious late-flowering mutant, we wondered whether its delayed leaf senescence is associated with a delayed plant development. To minimize a possible influence of plant development on leaf senescence, the detached rosette leaves of 40-day-old Col-0 and *ref6-1* plants (whole plants were still in vegetative growth) under short day-growth conditions were dark treated for 4 days to examine their dark-induced senescence phenotypes. It was found that the leaves of *ref6-1* plants exhibited an obvious stay-green phenotype compared to those of Col-0 (Fig 7A). The measurements of Chl content (Fig 7B) and Fv/Fm ratio (Fig 7C) were consistent with the phenotypic observations. Similar results were obtained on 4 DAD with the rosette leaves of 14-day-old plants under long day-growth conditions (Fig 7D–7F), implying that the stay-green phenotype of *ref6-1* is independent of plant development. To manifest a direct role of REF6 in promoting leaf senescence, a dexamethasone (DEX)-induced expression of *REF6* was designed and performed. Rosette leaves of 25-day-old *ref6-1*+*P*_*iDEX*_::*REF6* transgenic plants (under long day-growth conditions) were treated with 30 μM chemical inducer DEX, and a stronger yellowing phenotype was observed on 3 DAD, as compared with those of un-induced controls (Fig 7G–7I and S2C Fig). These results convincingly demonstrate that REF6 directly promotes leaf senescence independently of plant development.

## Discussion

### REF6-catalyzed H3K27me3 demethylation is involved in the regulation of leaf senescence

Epigenetic modifications, especially histone methylations, have been implicated in the regulatory process of leaf senescence [19, 65, 66]. During leaf senescence, genes showing an increase in H3K4me3 mark are up-regulated, while those showing a decrease in H3K4me3 mark are down-regulated. Interestingly, for H3K27me3 modification, the trends are just opposite [65, 66]. A previous report specifically revealed that the early-senescence activation of *WRKY53*, a key regulatory gene of leaf senescence, occurs concomitantly with a significant increase in active H3K4 marks but without a significant change in inactive H3K27 marks at its 5’ end and coding regions [19]. Just immediately before our resubmission, an online paper showed that an H3K4 specific demethylase, JMJ16, is apparently involved in this process by demethylating H3K4 at *WRKY53* as well as *SAG201* to prevent precocious leaf senescence in mature leaves [67]. Intriguingly, the ectopic overexpression of *SUVH2*, a histone methyltransferase gene, significantly impaired the increase in H3K4 marks at both its 5’ end and coding regions but caused a significant increase in H3K27 marks at its 5’ end, repressing its transcription and consequently delaying leaf senescence [20]. These findings suggest a kind of the involvement of histone methylations in the regulation of leaf senescence. Nevertheless, very little is known about the *in vivo* details of their involvement in the regulation of developmental leaf senescence. In this study, we identified an H3K27me3 demethylase, REF6, as a direct transcriptional regulator of *NYE1*/*2*, which is responsible for catalyzing Chl degradation during leaf senescence. Further analyses demonstrated that REF6 is also directly involved in the transcriptional regulation of major senescence regulatory and functional genes, which mediate ethylene signaling (*EIN2*, *ORE1*, and *NAP*) [51], abscisic acid/abiotic stress signaling (*AtNAC3* and *NTL9*) [68, 69], jasmonic acid (JA) biosynthesis (*LOX1*) [70], salicylic acid biosynthesis/signaling (*PAD4*) [71], and nitrogen remobilization (*PPDK*) [72] during leaf senescence. Consistently, as leaves age, REF6 reduces H3K27me3 level at all the ten genes. The loss-of-function mutation or ectopic overexpression of *REF6* significantly alters the initiation/progression dynamics of both Chl degradation and the general leaf senescence. Notably, REF6-upreglated Chl degradation and leaf senescence is independent of the developmental process of the whole plant. Based on the above findings, we for the first time reveal the involvement of a methylation status regulator, REF6, in the regulation of both Chl degradation and the general leaf senescence process.

H3K27 methylation is an important epigenetic modification involved in the regulation of gene expressions. In *Arabidopsis*, a genome-wide profiling identified 10 to 20% of genes that are marked by H3K27me3, depending on plant organs or their developmental states [2, 73]. The absence of REF6 incurs a genome-wide H3K27me3 hypermethylation, implying that REF6 might regulate a variety of growth and developmental processes [11]. Compared with those in 10-day-old seedlings, significant decreases in H3K27me3 level at all the ten genes in the 40-day-old leaves of *ref6-1* suggest that REF6 may not be the only demethylase responsible for H3K27me3 demethylation as leaves age. To check whether ELF6, another identified H3K27me3 demethylase, is possibly involved in this process, the dark-induced senescence phenotypes of *elf6-5* and *ref6-1 elf6-5* were examined four days after treatment in darkness (S5 Fig). It was found that the *elf6-5* mutant showed a similar senescence phenotype to that of Col-0, and no obvious differences were detected in their senescence phenotype between *ref6-1 elf6-5* double mutant and *ref6-1* single mutant. The observations suggest that ELF6 might not play a substantial role in regulating Chl degradation and leaf senescence, which is reminiscent of that ELF6 also plays a role different from that of REF6 in regulating flowering [14, 74]. Further efforts are needed to identify additional demethylase(s) responsible for H3K27me3 demethylation during the aging of leaves.

### *REF6* represents a new catalog of *SAGs*

*SAGs* are generally identified by their elevated transcription during senescence. We measured the relative transcript levels of *REF6* in the different tissues of Col-0 plants, and found that *REF6* had a high transcript level in the flower and capsule (S6A Fig). We also measured its relative transcript levels during the aging of leaves and during dark treatment. The transcription of *REF6* increased but not greatly from day 10 to day 25, even more gently from day 25 to day 40 (S1 Fig); similarly, during dark-induced senescence, the transcription of *REF6* elevated only slightly (S6B Fig). These measurements suggest that *REF6* is not a typical kind of *SAGs* in this regard, but represents a new catalog of *SAGs* that act to up-regulate the expression of other *SAGs*, likely through enriching their protein abundance or enhancing their enzymatic activity during senescence. As an enzyme, REF6 was once proposed to be recruited by NF-YA and BES1 onto its targets [15, 16]. Surprisingly, it was recently revealed that REF6 could directly bind to the CTCTGYTY motif of its targets via its zinc finger domain [17, 18], with *CUC1* and *PIN 1*/*3*/*7* being subsequently reported regulated as such [17, 75]. Here we demonstrate that the binding of REF6 to its targets could be mediated by the CTCTGYTY motifs located not only in the promoters but also in the coding regions (*EIN2*, *ORE1*, *NAP*, *PPDK*, *PAD4*, *LOX1*, and *NTL9*) of *SAGs*.

### H3K27me3 methylation is an epigenetic mechanism hindering the premature transcriptional activation of key *SAGs*

**Fig 8:**
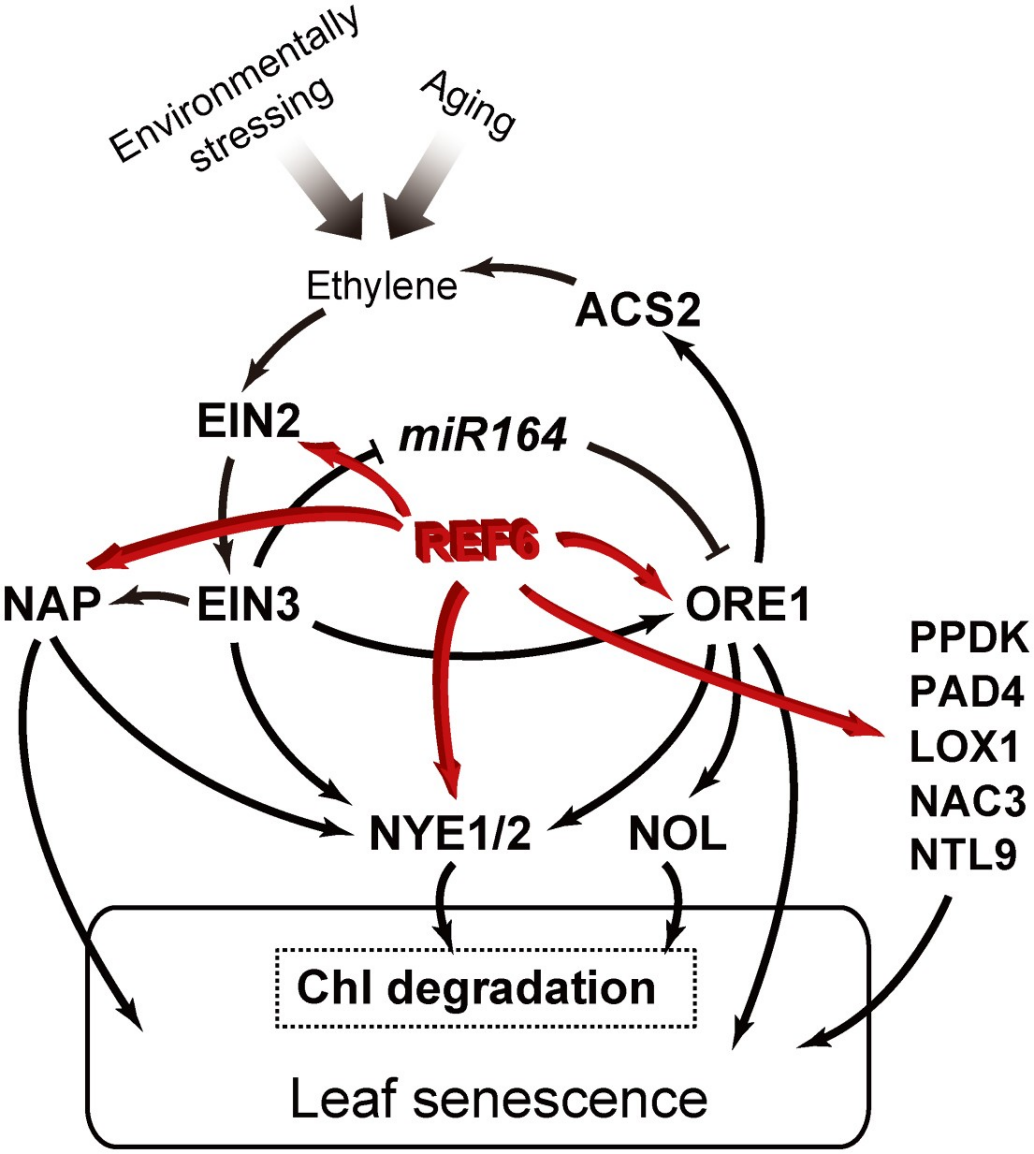
Major target *SAGs* of REF6 in the regulation of leaf senescence. ORE1 and NAP, acting downstream of EIN2 and EIN3, are the two major NAC transcription factors responsible for promoting Chl degradation and leaf senescence by up-regulating the expression of *NYE1/2* as well as other *SAGs*. EIN3 is also involved in a feed-forward regulation by directly suppressing the expression of *miR164*, which targets *ORE1* at the post-transcriptional level [46, 49, 51–54]. In this study, it is revealed that, during leaf senescence, REF6 directly facilitates the activation of the whole pathway by reducing the H3K27me3 level at *EIN2*, *ORE1*, and *NAP* [51] as well as A*tNAC3*, *NTL9*, L*OX1*, *PAD4* and *PPDK* [68–72].

Leaf senescence is initiated with a genome-wide transcriptional reprogramming [63, 76], and a large number of transcriptionally-enhanced *SAGs* have been identified over the last decade or so, some of which were found to function as modules [26, 77–79]. Nevertheless, a fundamental question remains unanswered, i.e. by which mechanism(s) these *SAGs* are kept transcriptionally silenced before the initiation of senescence. In this study, we show that REF6 directly upregulates ten major *SAGs* and is responsible for their H3K27me3 demethylation during the aging process of leaves (Fig 6 and S4 Fig), and importantly, loss-of-function of *REF6* represses their transcription, whereas overexpression of *REF6* enhances their transcription (Figs 2A, 4D and 5A and S1–S3 Tables), consequently causing a change in the dynamics of leaf senescence initiation (Fig 2C–2F). *EIN2*, *ORE1*, and *NAP*, interconnected with *EIN3* and *miR164*, form the framework of a core regulatory module (Fig 8) primarily responsible for the regulation of developmental leaf senescence as well as Chl degradation [46, 49, 51, 52]. Meanwhile, we found that *ref6-1* mutant showed insensitivity to ethylene treatment compared with Col-0 (S7 Fig). Our findings suggest that H3K27me3 methylation is an epigenetic mechanism hindering the premature transcriptional activation of key *SAGs* activated by major phytohormones’ and stresses’ signaling, ethylene signaling in particular. Our findings help to explain the “aging effect” on senescence induction [55, 56]. The scope and extent of H3K27me3 methylation as a prohibiting mechanism to other *SAGs*’ premature transcriptions need to be further investigated.

## Materials and methods

### Plant materials and growth conditions

All mutants and transgenic lines were derived from *Arabidopsis thaliana* ecotype Columbia (Col-0) unless stated otherwise. Generation and identification of *nye1 nye2* were carried out as described previously [39, 58]. The *ref6-1*, *elf6-5*, *ref6-3 ef6-5* mutants and *ref6-1*+*P*_*REF6*_::*REF6-HA* plants were kindly provided by Dr. Xiaofeng Cao (Chinese Academy of Sciences, Beijing). To generate *nye1-1*+*P*_*iDEX*_::*REF6*, *ref6-1*+*P*_*iDEX*_::*REF6*, and *ref6-1*+*P*_*iDEX*_::*NYE1* transgenic lines, the full-length coding sequences (CDS) of *REF6* and *NYE1* were PCR amplified and cloned into the vector p*TA7002*, respectively. The resultant constructs were introduced into *nye1-1* and *ref6-1* mutant plants, respectively, by using the floral-dipping method.

Seeds were germinated in soil and plants were grown at 23 °C under 16-h light/8-h dark for long day conditions or 16-h dark/8-h light for short day conditions in a growth room equipped with cool-white fluorescent lights (90–100 μmol m^-2^ s^-1^) unless indicated otherwise. The 3rd and 4th rosette leaves from 25-day-old soil-grown plants were incubated in complete darkness as described previously [39].

### Y1H screening

Y1H screening is performed with the Matchmaker Gold Yeast One-Hybrid Library Screening System (Clontech). The bait fragment (the -532 bp fragment of *NYE1* promoter, [57]) was amplified by PCR and cloned into the p*AbAi* vector. The resultant vector was subsequently linearized and introduced into the yeast strain Y1H Gold to generate a bait-reporter strain, which was then used to screen a cDNA library generated from detached leaves incubated in darkness for 12 h. Approximately 5×10^5^ transformants were initially screened out on plates containing SD/-Leu media supplemented with 100 ng/ml *Aureobasidin A*. Prey fragments were identified from the positive colonies by DNA sequencing. For re-transformation assay, the full-length coding sequence of *REF6* was amplified from Col-0 cDNA by use of gene-specific primers (S4 Table). The PCR products were then cloned into the p*GADT7* (Clontech) prey vector and the resultant vectors were subsequently transferred into the previously mentioned bait-reporter yeast strain.

### RNA isolation and quantitative RT-PCR

Total RNAs were extracted by using Trizol reagent (Invitrogen), and DNA remnants were removed by Dnase I (Takara) treatment. First-strand cDNAs were synthesized with the PrimeScript RT reagent kit (Takara) and used as templates for quantitative RT-PCR (qRT-PCR) with SYBR Premix Ex Taq TM (Takara). The qPCR analyses were carried out with the MyiQ5 Real Time PCR Detection System (Bio-Rad, Hercules, CA). *ACTIN2* (*AT3g18780*) was used as an internal reference to normalize the qPCR data. Gene-specific primers are listed in S4 Table.

### RNA-sequencing and data analysis

Total RNA of the whole seedlings or detached leaves was extracted by using a Trizol kit (Takara). Fifty bp single end RNA-sequencing was conducted by using the BGISEQ-500 platform established by Beijing Genomics Institute, and the reads were aligned by using Bowtie 2 instead of Bowtie [80]. A gene with a cut-off value of two-fold change and p-value less than 0.01 was defined as a differentially expressed gene. R program “princomp” was used to conduct PCA analysis. Heatmap.2 in the ‘gplots’ package of R program was used for the construction of heat maps.

### Electrophoretic mobility shift assay (EMSA)

An *REF6C* (encoding amino acids 1,239–1,360) fragment was cloned into p*GEX-6p-1*, and GST-REF6C recombinant fusion protein and GST protein were then expressed in *Escherichia coli* (BL21 codon plus, Stratagene) and purified with glutathione sepharose 4B beads (GE Healthcare). Biotin-labeled DNA probes are listed in S4 Table. Unlabeled competitors were added in 400-fold molar excess. EMSA is carried out with the Light Shift Chemiluminescent EMSA Kit (Thermo Scientific). 20 μl reaction mixture contained 2 μl binding buffer, 0.3 μl poly (dI-dC), 4 μg purified fusion protein and 1 μl biotin-labeled annealed oligonucleotides. After incubation at room temperature for 30 min, the reaction mixture was electrophoresed on a 5% polyacrylamide mini-gel (containing 3% glycerol), then transferred onto a positively charged nylon membrane (Amersham Biosciences), which was illuminated by use of an ultraviolet lamp for cross-linking. Biotin-labeled DNA was detected with Pierce chemiluminescence kit (Thermo Scientific).

### Chromatin immunoprecipitation (ChIP) assay

ChIP assay was performed as described previously [17] with slight modifications. For measuring REF6 enrichments: chromatins were isolated from about 2 g of formaldehyde cross-linked rosette leaves from 10-day-old transgenic plants and Col-0 plants. For the determination of H3K27me3 levels, chromatins were isolated from about 2 g of formaldehyde cross-linked rosette leaves of 10-day-old seedlings and 40-day old leaves of Col-0 and *ref6-1* plants, respectively, which were then sonicated to produce 0.2- to 1-kb DNA fragments with a Branson sonicator (40-s bursts at -88 watts). The lysates were diluted 10-fold with ChIP dilution buffer to decrease the concentration of SDS to 0.1%, which was then cleared by centrifugation (16,000 g for 15 min at 4 °C). After 5% being taken out and used as input, the rest supernatant was incubated with the antibodies for HA (Sigma, H6908) or H3K27me3 (Millipore, 07–449) overnight at 4 °C. Chromatin was collected by using Protein A/G magnetic beads, then washed, eluted, and reverse cross-linked, and DNA purification was then performed. DNA fragments were purified by using the ChIP-qPCR purification kit (Zymo Research). The purified DNA was re-suspended in double-distilled water, and enriched DNA fragments were quantified by qPCR with the primers listed in S4 Table. Input samples were reverse–cross-linked and used to normalize the qPCR data for each ChIP sample.

### Ethylene treatment

Ethylene treatment was carried out principally as reported [81]. Ethephon was purchased from Shanghai Sangon Biotechnology Co., and leaves were treated in an air-tight container (desiccator). A1 Methephon stock solution was prepared with ethanol, and 86.5 μl of the stock solution were added to 200 ml of 5 mM Na_2_HPO_3_ placed in a 17.3 L desiccator to create the 5 μM air concentration of ethylene. The cover was closed immediately after addition, and the desiccator was placed under light or in the dark according to the requirement of the experimental design.

### Measurements of Ion leakage rates

Before or after a 4-day treatment in the dark, the detached leaves were incubated in deionized water for at least 4 h (< 10 h), and small fractions of the elution water solutions were subsequently taken out for the initial value determination (C1). The leaf samples were then boiled in the same deionized water for 15 min. After cooling, the elution water solutions were determined again (C2) [49]. The ratio of C1: C2 was calculated as the percentage of ion leakage. We used 3 ml deionized water for one leaf measurement in a 5-ml centrifuge tube.

### Measurements of Chl contents and Fv/Fm ratios

Chl contents were measured by using SPAD-502 PLUS, and maximal photochemical efficiencies of PSII (Fv/Fm) were measured with LI-COR6400 (http://www.licor.com/env/products/photosynthesis) according to manufacturer’s instructions.

### Statistical analysis

Data are given as mean ± SD and were analyzed by Student’s *t* test or one-way ANOVA.

### Accession numbers

*NYE1* (*AT4G22920*), *NYE2* (*AT4G11910*), *EIN2* (*AT5G03280*), *ORE1*/*NAC2*/*NAC092* (*AT5G39610*), *NAP* (*AT1G69490*), *REF6* (*AT3G48430*), *ACTIN2* (*AT3G18780*), *UBQ* (*AT4G05320*), *GPAT4* (*AT1G01610*), *NAC004* (*AT1G02230*), *TUB* (*AT5G62690*), *PAD4* (*AT3G52430*), *PPDK* (*AT4G15530*), *LOX1* (AT1G55020), *NAC3* (AT3G15500), *NTL9* (AT4G35580). RNA-seq data obtained in this study were deposited at the NCBI short read archive under Bioproject identifier PRJNA518728 with accession number: SRR8518110 to SRR8518121.

## Supporting information

S1 Fig Transcript levels of *REF6* in Col-0, *ref6-1*, and *ref6-1*+*P*_*REF6*_::*REF6-HA* plants on the dates indicated. (A) Abundances of semi-quantitative RT-PCR products of *REF6* in the leaves detached from 10-day-old or 40-day-old Col-0, *ref6-1*, and *ref6-1*+*P*_*REF6*_::*REF6-HA* plants grown under long day-growth conditions. (B) Relative transcript levels of *REF6* in the leaves detached from 10-day-old, 25-day-old, or 40-day-old Col-0, and *ref6-1*+*P*_*REF6*_::*REF6-HA* plants grown under long day-growth conditions. Data are mean ± SD (n = 3). *P < 0.05, ***P < 0.001 by paired Student’s *t* test.
(DOCX)

S2 Fig Induced transcript levels of *REF6* and *NYE1* in their overexpressed lines. (A) Relative transcript levels of *NYE1* in 25-day-old *ref6-1*+*P*_*iDEX*_::*NYE1* transgenic plants grown under long day-growth conditions with or without DEX treatment. -1, -3, and -4 represent different transgenic lines. (B) Relative transcript levels of *REF6* in 25-day-old *nye1-1*+*P*_*iDEX*_::*REF6* transgenic plants under long day-growth conditions with or without DEX treatment. -1, -2, and -5 represent different transgenic lines. (C) Relative transcript levels of *REF6* in 25-day-old *ref6-1*+*P*_*iDEX*_::*REF6* plants under long day-growth conditions with or without DEX treatment. -2, -3, and -5 represent different transgenic lines. In (A)-(C), data are mean ± SD (n = 3), **P < 0.01, ***P < 0.001 by paired Student’s *t* test.
(DOCX)

S3 Fig Fv/Fm ratios of the rosette leaves of *REF6* mutant and overexpressed plants. Fv/Fm ratios were determined in the 25-day and 40-day old rosette leaves of *ref6-1* and *ref6-1*+*P*_*REF6*_::*REF6-HA* plants as well as Col-0 and *nye1 nye2* plants. Data are mean ± SD (n = 10). *P < 0.05, **P < 0.01, ***P < 0.001 by paired Student’s *t* test.
(DOCX)

S4 Fig Loss-of-function of REF6 increases H3K27me3 levels at *PPDK*, *PAD4*, *LOX1*, *NAC3*, and *NTL9* genes. (A) Schematic diagrams of *PPDK*, *PAD4*, *LOX1*, *NAC3*, and *NTL9* genes and positions of the primers used for determining their H3K27me3 levels. Primer sequences are listed in S4 Table. (B) H3K27me3 levels of *PPDK*, *PAD4*, *LOX1*, *NAC3*, and *NTL9*, expressed as the percentage of input, in the 10-day-old seedlings of Col-0, *ref6-1*, and *ref6-1*+*P*_*REF6*_::*REF6-HA* grown under long day-growth conditions. (C) H3K27me3 levels of *PPDK*, *PAD4*, *LOX1*, *NAC3* and *NTL9* genes in the leaves detached from the 40-day-old plants of Col-0, *ref6-1*, and *ref6-1*+*P*_*REF6*_::*REF6-HA* grown under long day-growth conditions. In (B)—(C), data are mean ± SD (n = 3). *P < 0.05, **P < 0.01, ***P < 0.001 by paired Student’s *t* test.
(DOCX)

S5 Fig Loss-of-function of ELF6 causes no effect on leaf senescence. (A) Senescence phenotypes of the leaves of indicated genotypes on 4 DAD. Leaves were detached from 25-day-old plants under long day-growth conditions. (B, C) Chl contents (B) and Fv/Fm ratios (C) in the leaves shown in (A). Data are mean ± SD (n = 10). Marking with different letters means a statistical significance at P < 0.05 by one-way ANOVA test.
(DOCX)

S6 Fig Relative transcript levels of *REF6* in the different tissues of Col-0 plants or in the leaves of Col-0 plants after dark treatment. (A) Relative transcript levels of *REF6* in the different tissues of Col-0 plants. (B) Relative transcript levels of *REF6* in the leaves of Col-0 plants after dark treatment. In (A) and (B), data are mean ± SD (n = 3). Marking with different letters means a statistical significance at P < 0.05 by one-way ANOVA test.
(DOCX)

S7 Fig Effects of ethylene treatment on *REF6* mutant and overexpressed plants. (A) Senescence phenotypes of the attached leaves of 25-day-old Col-0, *ref6-1*, and *ref6-1*+*P*_*REF6*_::*REF6-HA* plants two days after ethylene treatment. (B) Senescence phenotypes of the leaves detached from 25-day-old Col-0 and *ref6-1* plants two days after ethylene treatment. (C) Chl contents in the leaves shown in (A). (D) Chl contents in the leaves shown in (B). Marking with different letters means a statistical significance at P < 0.05 by one-way ANOVA test.
(DOCX)

S1 Table Differently expressed genes between Col-0 and *ref6-1*. (XLSX)

S2 Table Data for making heat maps. (XLSX)

S3 Table Down-regulated genes in the 40-day-old leaves of *ref6-1*. (XLSX)

S4 Table Primers used in this study. (XLS)
