# Supplementary material for: The H3K27me3 demethylase REF6 promotes leaf senescence through directly activating major senescence regulatory and functional genes in Arabidopsis
Source: PLoS Genet. 2019 Apr 10;15(4):e1008068. doi: 10.1371/journal.pgen.1008068 (PMC6457497; doi:10.1371/journal.pgen.1008068)

**S1 Fig. Transcript levels of *REF6* in Col-0, *ref6-1*, and *ref6-1*+*P_REF6_*::*REF6-HA* plants on the dates indicated.** (A) Abundances of semi-quantitative RT-PCR products of *REF6* in the leaves detached from 10-day-old or 40-day-old Col-0, *ref6-1*, and *ref6-1*+*P_REF6_*::*REF6-HA* plants grown under long day-growth conditions. (B) Relative transcript levels of *REF6* in the leaves detached from 10-day-old, 25-day-old, or 40-day-old Col-0, and *ref6-1*+*P_REF6_*::*REF6-HA* plants grown under long day-growth conditions. Data are mean ± SD (n=3). *P < 0.05, ***P < 0.001 by paired Student’s *t* test.


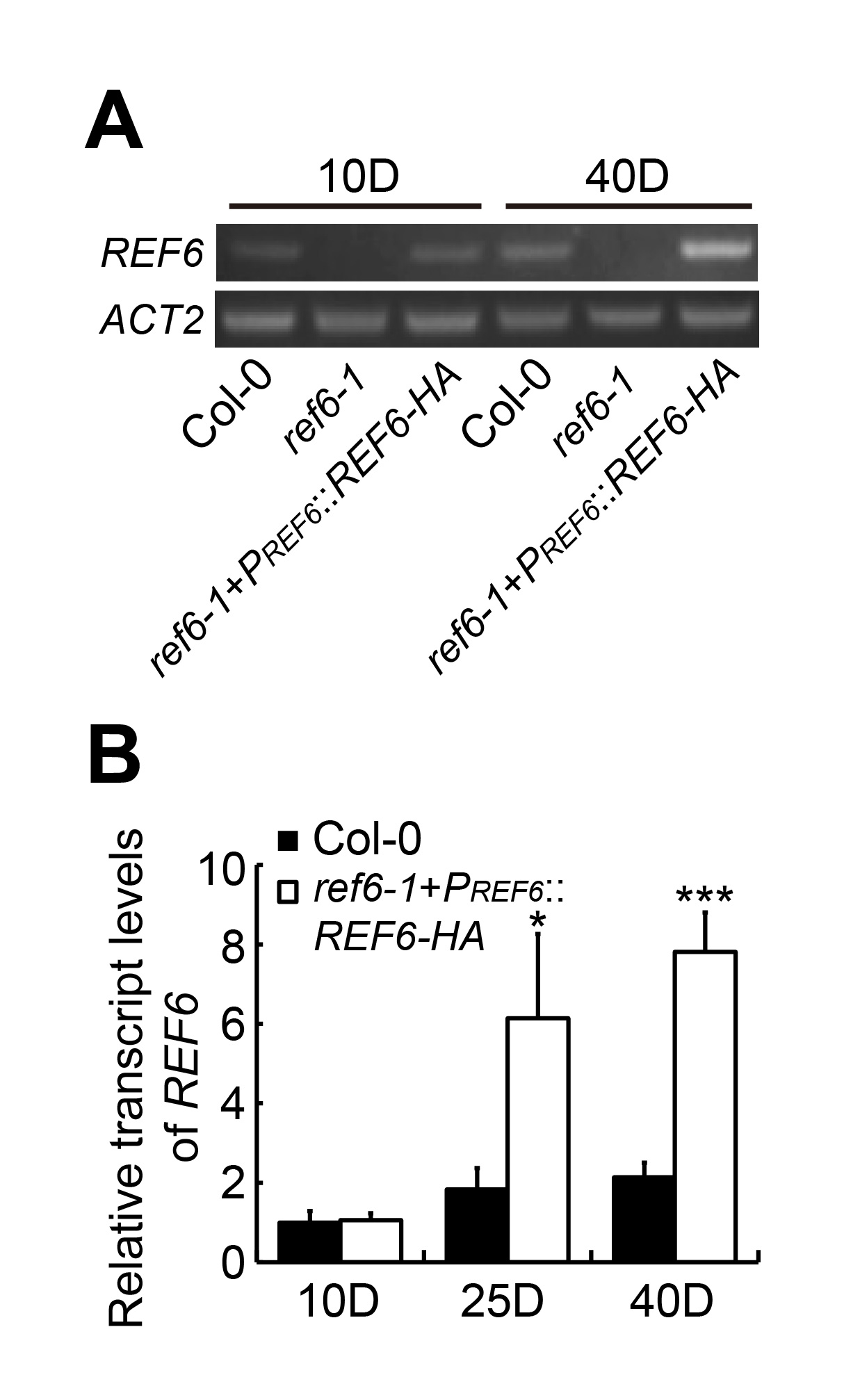

Supplement: S1 Fig — (A) Abundances of semi-quantitative RT-PCR products of REF6 in the leaves detached from 10-day-old or 40-day-old Col-0, ref6-1, and ref6-1+PREF6::REF6-HA plants grown under long day-growth conditions. (B) Relative transcript levels of REF6 in the leaves detached from 10-day-old, 25-day-old, or 40-day-old Col-0, and ref6-1+PREF6::REF6-HA plants grown under long day-growth conditions. Data are mean ± SD (n = 3). *P < 0.05, ***P < 0.001 by paired Student’s t test. (DOCX) [file pgen.1008068.s001.docx]
