# Supplementary material for: The H3K27me3 demethylase REF6 promotes leaf senescence through directly activating major senescence regulatory and functional genes in Arabidopsis
Source: PLoS Genet. 2019 Apr 10;15(4):e1008068. doi: 10.1371/journal.pgen.1008068 (PMC6457497; doi:10.1371/journal.pgen.1008068)

**S2 Fig. Induced transcript levels of *REF6* and *NYE1* in their overexpressed lines.** (A) Relative transcript levels of *NYE1* in 25-day-old *ref6-1*+*P_iDEX_*::*NYE1* transgenic plants grown under long day-growth conditions with or without DEX treatment. -1, -3, and -4 represent different transgenic lines. (B) Relative transcript levels of *REF6* in 25-day-old *nye1-1*+*P_iDEX_*::*REF6* transgenic plants under long day-growth conditions with or without DEX treatment. -1, -2, and -5 represent different transgenic lines. (C) Relative transcript levels of *REF6* in 25-day-old *ref6-1*+*P_iDEX_*::*REF6* plants under long day-growth conditions with or without DEX treatment. -2, -3, and -5 represent different transgenic lines. In (A)-(C), data are mean ± SD (n=3), **P < 0.01, ***P < 0.001 by paired Student’s *t* test.


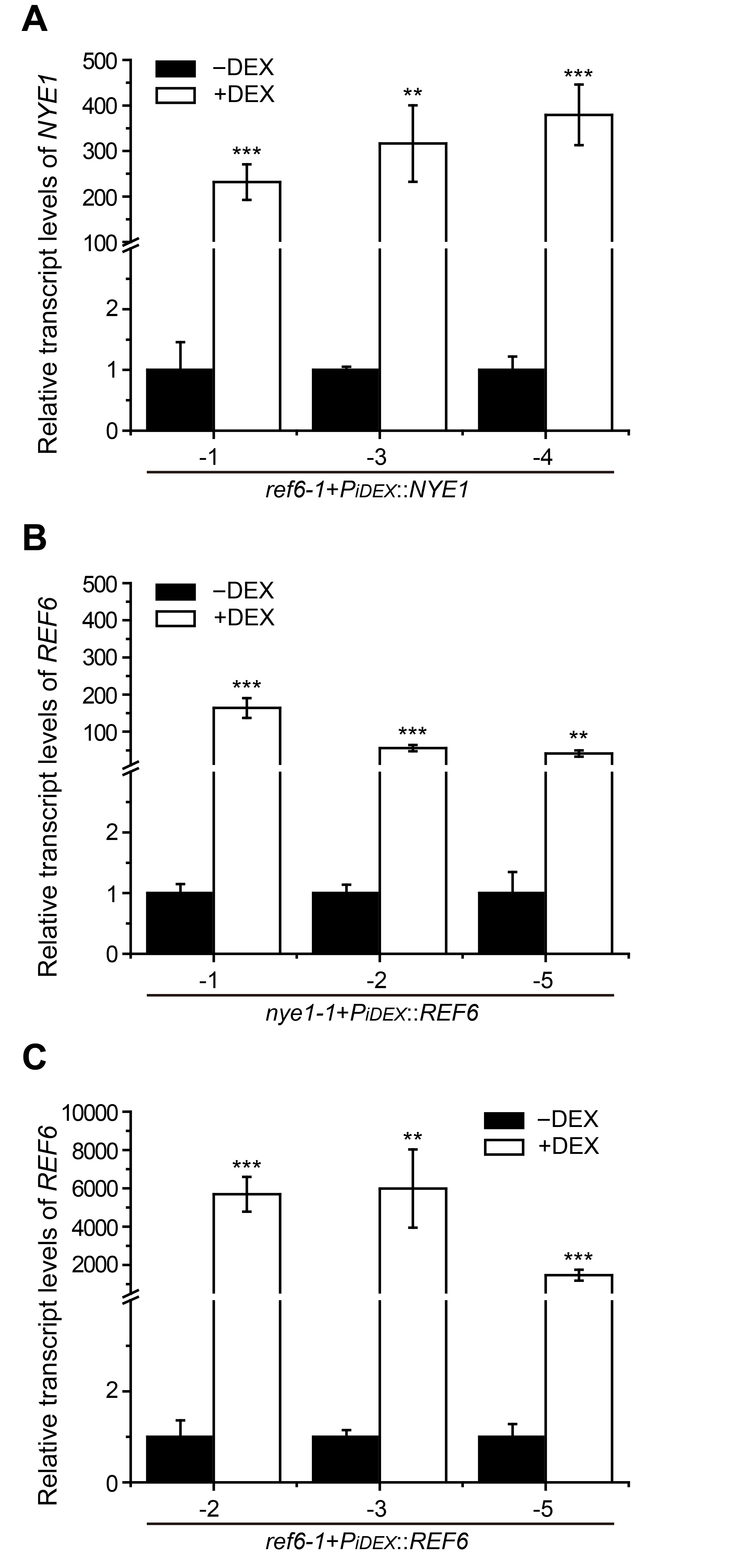

Supplement: S2 Fig — (A) Relative transcript levels of NYE1 in 25-day-old ref6-1+PiDEX::NYE1 transgenic plants grown under long day-growth conditions with or without DEX treatment. -1, -3, and -4 represent different transgenic lines. (B) Relative transcript levels of REF6 in 25-day-old nye1-1+PiDEX::REF6 transgenic plants under long day-growth conditions with or without DEX treatment. -1, -2, and -5 represent different transgenic lines. (C) Relative transcript levels of REF6 in 25-day-old ref6-1+PiDEX::REF6 plants under long day-growth conditions with or without DEX treatment. -2, -3, and -5 represent different transgenic lines. In (A)-(C), data are mean ± SD (n = 3), **P < 0.01, ***P < 0.001 by paired Student’s t test. (DOCX) [file pgen.1008068.s002.docx]
