# Supplementary material for: The H3K27me3 demethylase REF6 promotes leaf senescence through directly activating major senescence regulatory and functional genes in Arabidopsis
Source: PLoS Genet. 2019 Apr 10;15(4):e1008068. doi: 10.1371/journal.pgen.1008068 (PMC6457497; doi:10.1371/journal.pgen.1008068)

**S3 Fig. Fv/Fm ratios of the rosette leaves of *REF6* mutant and overexpressed plants.** Fv/Fm ratios were determined in the 25-day and 40-day old rosette leaves of *ref6-1* and *ref6-1*+*P_REF6_*::*REF6-HA* plants as well as Col-0 and *nye1 nye2* plants*.* Data are mean ± SD (n=10). *P < 0.05, **P < 0.01, ***P < 0.001 by paired Student’s *t* test.


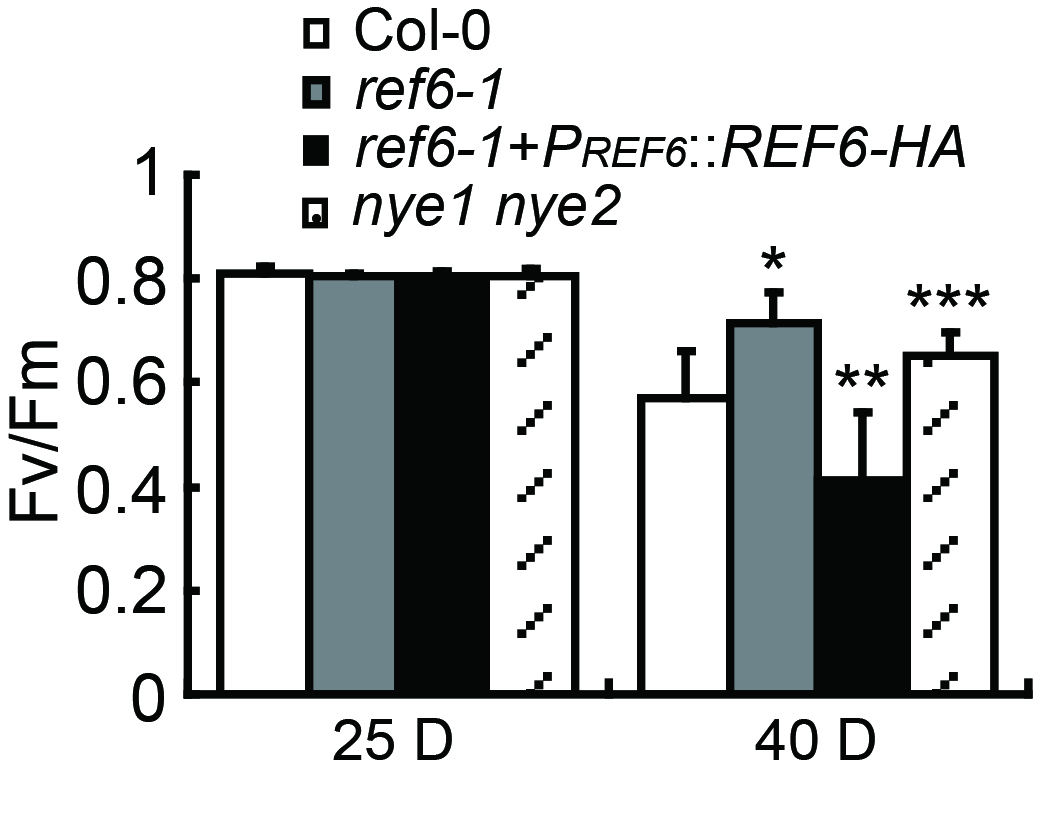

Supplement: S3 Fig — Fv/Fm ratios were determined in the 25-day and 40-day old rosette leaves of ref6-1 and ref6-1+PREF6::REF6-HA plants as well as Col-0 and nye1 nye2 plants. Data are mean ± SD (n = 10). *P < 0.05, **P < 0.01, ***P < 0.001 by paired Student’s t test. (DOCX) [file pgen.1008068.s003.docx]
