# Supplementary material for: The H3K27me3 demethylase REF6 promotes leaf senescence through directly activating major senescence regulatory and functional genes in Arabidopsis
Source: PLoS Genet. 2019 Apr 10;15(4):e1008068. doi: 10.1371/journal.pgen.1008068 (PMC6457497; doi:10.1371/journal.pgen.1008068)

**S4 Fig. Loss-of-function of REF6 increases H3K27me3 levels at *PPDK*, *PAD4*, *LOX1,* *NAC3* and *NTL9* genes.** (A) Schematic diagrams of *PPDK*, *PAD4*, *LOX1*, *NAC3*, and *NTL9* genes and positions of the primers used for determining their H3K27me3 levels. Primer sequences are listed in S4 Table. (B) H3K27me3 levels of *PPDK*, *PAD4*, *LOX1*, *NAC3*, and *NTL9*, expressed as the percentage of input, in the 10-day-old seedlings of Col-0, *ref6-1* and *ref6-1*+*P_REF6_*::*REF6-HA* grown under long day-growth conditions. (C) H3K27me3 levels of *PPDK*, *PAD4*, *LOX1*, *NAC3*, and *NTL9* genes in the leaves detached from the 40-day-old plants of Col-0, *ref6-1* and *ref6-1*+*P_REF6_*::*REF6-HA* grown under long day-growth conditions. In (B) - (C), data are mean ± SD (n=3). *P < 0.05, **P < 0.01, ***P < 0.001 by paired Student’s *t* test.


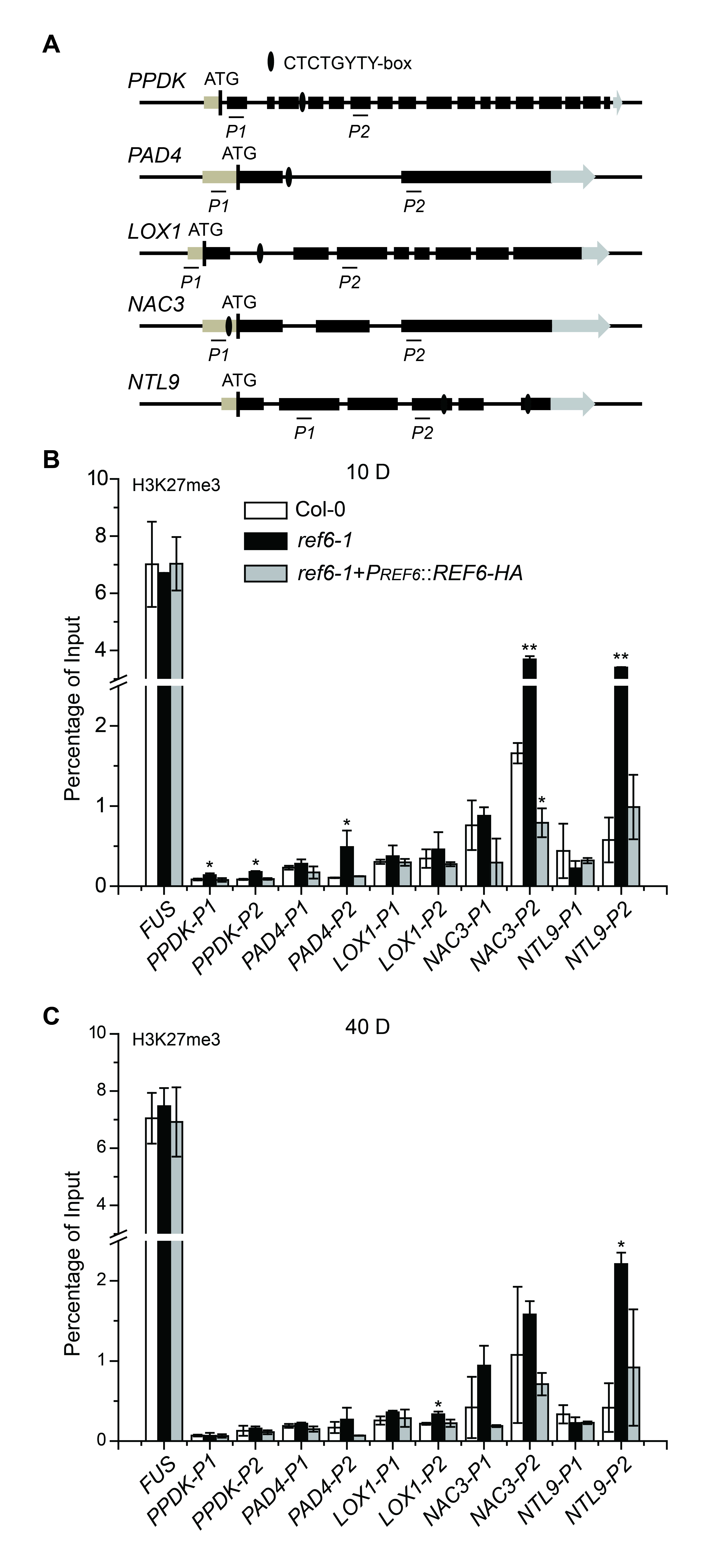

Supplement: S4 Fig — (A) Schematic diagrams of PPDK, PAD4, LOX1, NAC3, and NTL9 genes and positions of the primers used for determining their H3K27me3 levels. Primer sequences are listed in S4 Table. (B) H3K27me3 levels of PPDK, PAD4, LOX1, NAC3, and NTL9, expressed as the percentage of input, in the 10-day-old seedlings of Col-0, ref6-1, and ref6-1+PREF6::REF6-HA grown under long day-growth conditions. (C) H3K27me3 levels of PPDK, PAD4, LOX1, NAC3 and NTL9 genes in the leaves detached from the 40-day-old plants of Col-0, ref6-1, and ref6-1+PREF6::REF6-HA grown under long day-growth conditions. In (B)—(C), data are mean ± SD (n = 3). *P < 0.05, **P < 0.01, ***P < 0.001 by paired Student’s t test. (DOCX) [file pgen.1008068.s004.docx]
