# Supplementary material for: The H3K27me3 demethylase REF6 promotes leaf senescence through directly activating major senescence regulatory and functional genes in Arabidopsis
Source: PLoS Genet. 2019 Apr 10;15(4):e1008068. doi: 10.1371/journal.pgen.1008068 (PMC6457497; doi:10.1371/journal.pgen.1008068)

**S5 Fig. Loss-of-function of ELF6 causes no effect on leaf senescence.** (A) Senescence phenotypes of the leaves of indicated genotypes on 4 DAD. Leaves were detached from 25-day-old plants under long day-growth conditions. (B, C) Chl contents (B) and Fv/Fm ratios (C) in the leaves shown in (A). Data are mean ± SD (n=10). Marking with different letters means a statistical significance at P < 0.05 by one-way ANOVA test.


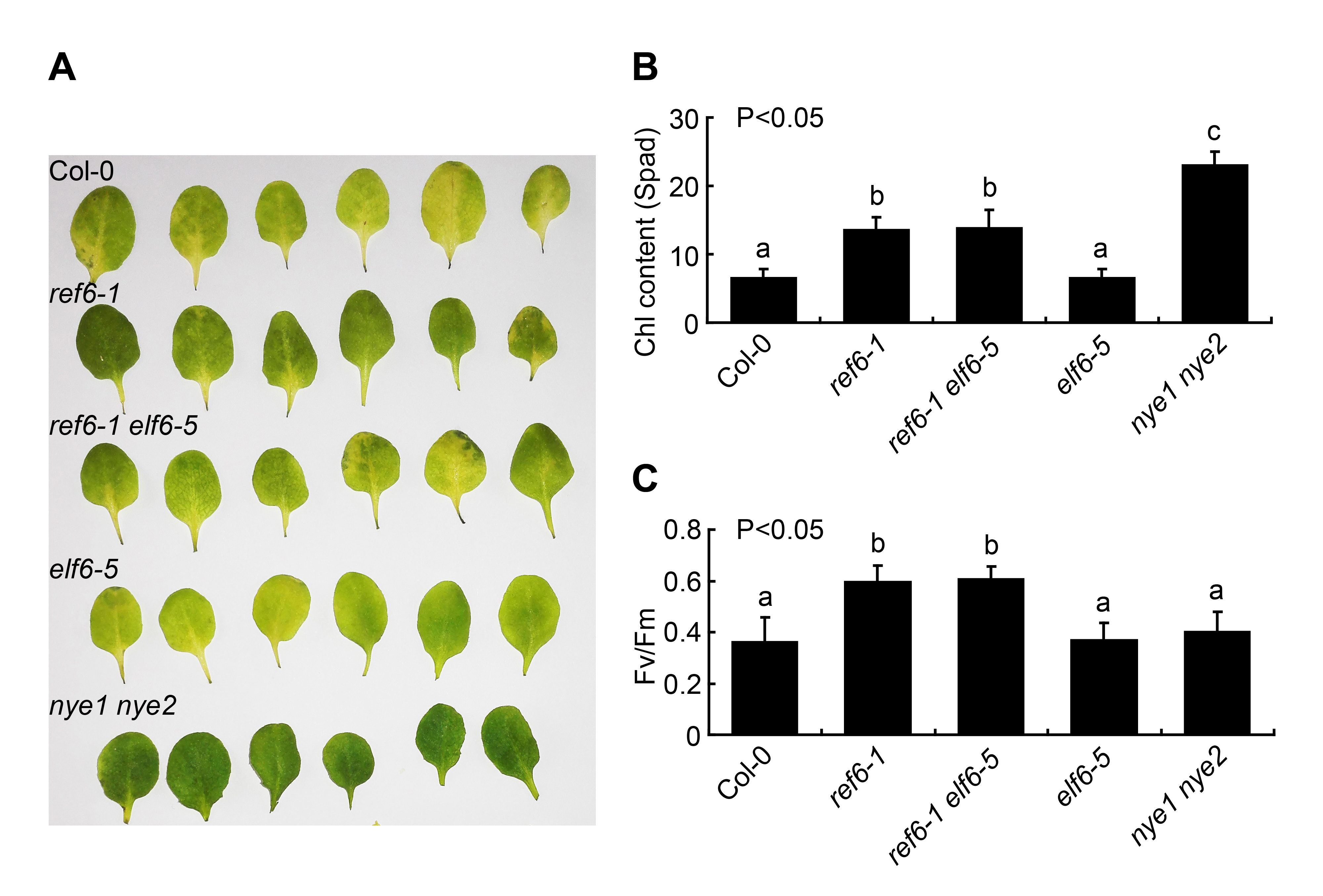

Supplement: S5 Fig — (A) Senescence phenotypes of the leaves of indicated genotypes on 4 DAD. Leaves were detached from 25-day-old plants under long day-growth conditions. (B, C) Chl contents (B) and Fv/Fm ratios (C) in the leaves shown in (A). Data are mean ± SD (n = 10). Marking with different letters means a statistical significance at P < 0.05 by one-way ANOVA test. (DOCX) [file pgen.1008068.s005.docx]
