# Supplementary material for: The H3K27me3 demethylase REF6 promotes leaf senescence through directly activating major senescence regulatory and functional genes in Arabidopsis
Source: PLoS Genet. 2019 Apr 10;15(4):e1008068. doi: 10.1371/journal.pgen.1008068 (PMC6457497; doi:10.1371/journal.pgen.1008068)

**S6 Fig. Relative transcript levels of *REF6* in the different tissues of Col-0 plants or in the leaves of Col-0 plants after dark treatment.** (A) Relative transcript levels of *REF6* in the different tissues of Col-0 plants. (B) Relative transcript levels of *REF6* in the leaves of Col-0 plants after dark treatment. In (A) and (B), data are mean ± SD (n=3). Marking with different letters means a statistical significance at P < 0.05 by one-way ANOVA test.


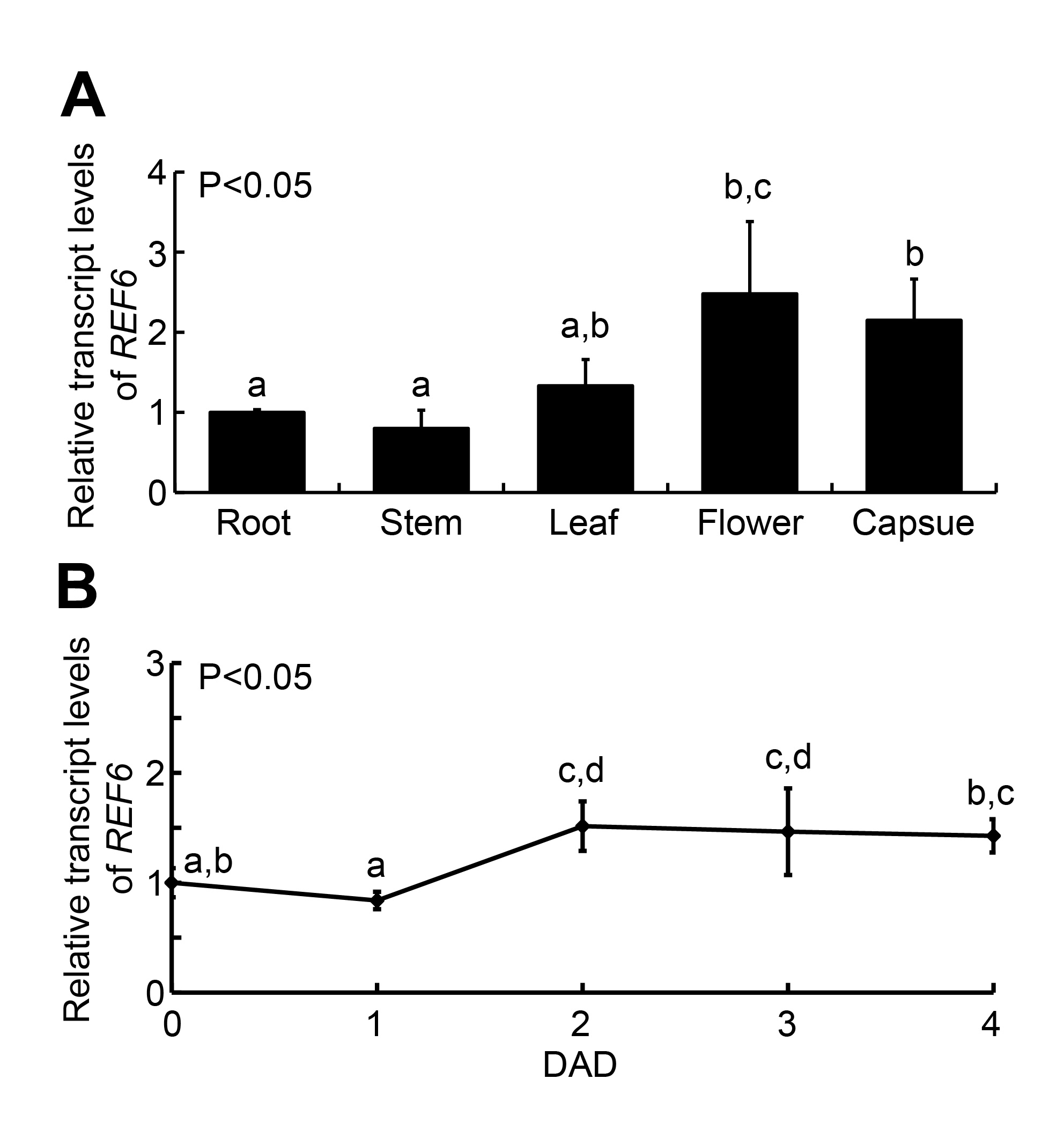

Supplement: S6 Fig — (A) Relative transcript levels of REF6 in the different tissues of Col-0 plants. (B) Relative transcript levels of REF6 in the leaves of Col-0 plants after dark treatment. In (A) and (B), data are mean ± SD (n = 3). Marking with different letters means a statistical significance at P < 0.05 by one-way ANOVA test. (DOCX) [file pgen.1008068.s006.docx]
