# Supplementary material for: The H3K27me3 demethylase REF6 promotes leaf senescence through directly activating major senescence regulatory and functional genes in Arabidopsis
Source: PLoS Genet. 2019 Apr 10;15(4):e1008068. doi: 10.1371/journal.pgen.1008068 (PMC6457497; doi:10.1371/journal.pgen.1008068)

**S7 Fig. Effects of ethylene treatment on *REF6* mutant and overexpressed plants.** (A) Senescence phenotypes of the attached leaves of 25-day-old Col-0, *ref6-1* and *ref6-1*+*P_REF6_*::*REF6-HA* plants two days after ethylene treatment. (B) Senescence phenotypes of the leaves detached from 25-day-old Col-0 and *ref6-1* plants two days after ethylene treatment. (C) Chl contents in the leaves shown in (A). (D) Chl contents in the leaves shown in (B). Marking with different letters means a statistical significance at P < 0.05 by one-way ANOVA test.


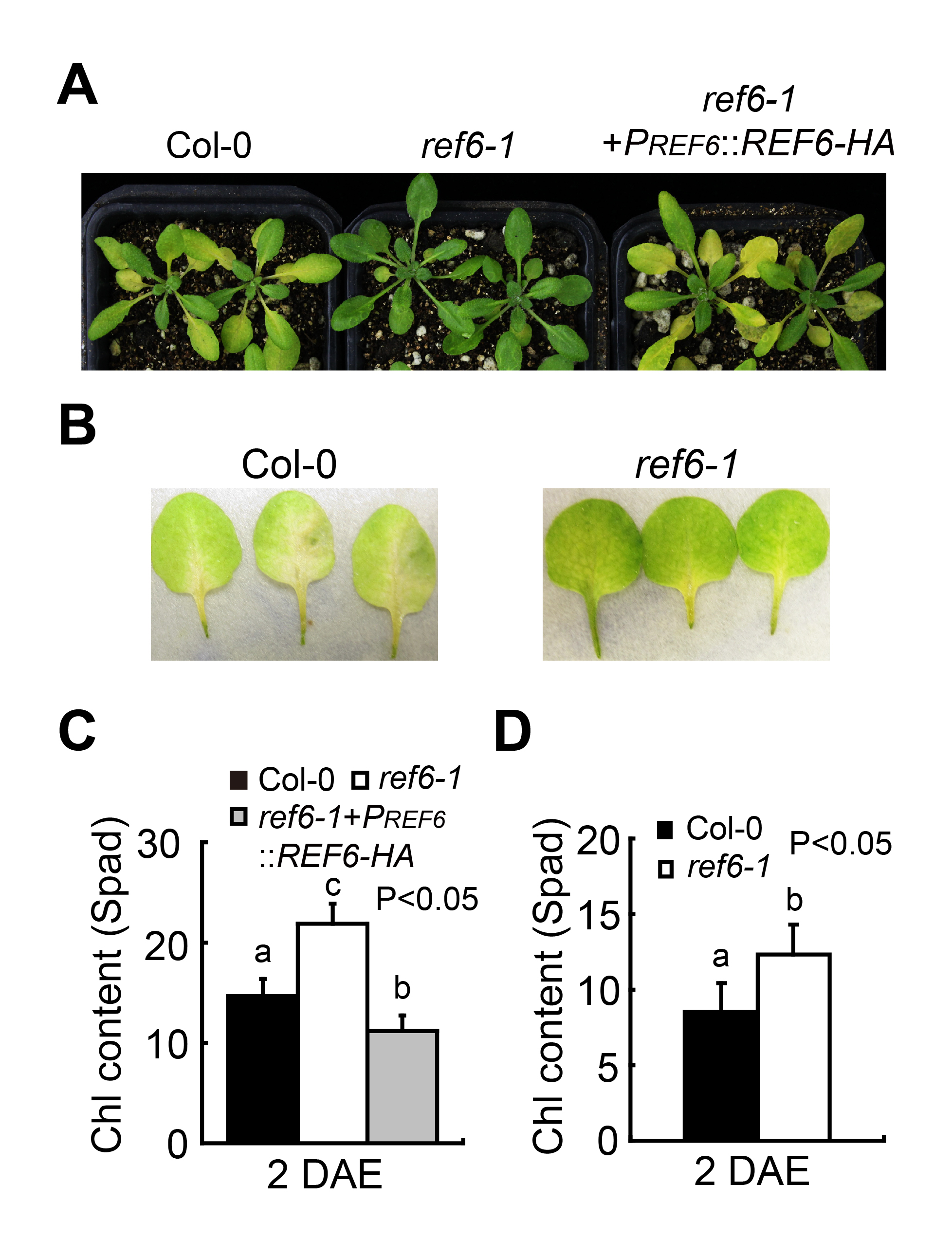

Supplement: S7 Fig — (A) Senescence phenotypes of the attached leaves of 25-day-old Col-0, ref6-1, and ref6-1+PREF6::REF6-HA plants two days after ethylene treatment. (B) Senescence phenotypes of the leaves detached from 25-day-old Col-0 and ref6-1 plants two days after ethylene treatment. (C) Chl contents in the leaves shown in (A). (D) Chl contents in the leaves shown in (B). Marking with different letters means a statistical significance at P < 0.05 by one-way ANOVA test. (DOCX) [file pgen.1008068.s007.docx]
